# Predicting the frequencies of drug side effects

**DOI:** 10.1038/s41467-020-18305-y

**Published:** 2020-09-11

**Authors:** Diego Galeano, Shantao Li, Mark Gerstein, Alberto Paccanaro

**Affiliations:** 1 Department of Computer Science, Centre for Systems and Synthetic Biology, Royal Holloway, University of London, Egham Hill, Egham, UK; 2 School of Applied Mathematics, Fundação Getulio Vargas, Rio de Janeiro, Brazil; 3 Department of Computer Science and Department of Biomedical Data Sciences, Stanford University, Stanford, CA USA; 4 Department of Molecular Biophysics and Biochemistry, Department of Computer Science, and Department of Statistics and Data Science, Yale University, New Haven, CT 06520 USA

**Keywords:** Computational models, Drug safety

## Abstract

A central issue in drug risk-benefit assessment is identifying frequencies of side effects in humans. Currently, frequencies are experimentally determined in randomised controlled clinical trials. We present a machine learning framework for computationally predicting frequencies of drug side effects. Our matrix decomposition algorithm learns latent signatures of drugs and side effects that are both reproducible and biologically interpretable. We show the usefulness of our approach on 759 structurally and therapeutically diverse drugs and 994 side effects from all human physiological systems. Our approach can be applied to any drug for which a small number of side effect frequencies have been identified, in order to predict the frequencies of further, yet unidentified, side effects. We show that our model is informative of the biology underlying drug activity: individual components of the drug signatures are related to the distinct anatomical categories of the drugs and to the specific drug routes of administration.

## Abstract (web summary)

Currently, the frequencies of drug side effects are determined in randomised controlled clinical trials. Here the authors develop an interpretable machine learning approach to predict the frequencies of unknown side effects for drugs with a small number of determined side effect frequencies.

## Introduction

The estimation of the frequencies of the side effects is crucial in drug risk–benefit^1^ assessment. Currently, these frequencies are estimated using intervention and placebo groups during randomised controlled trials. Although these trials are limited by sample size, time frame and lack of accrual^2^, they are the standard approach to eliminate selection bias in clinical medicine^3^.

However, it is well recognised that numerous side effects are not observed during clinical trials^4^ but are only identified after the drug has reached the market^5–7^. For this reason, drug side effects remain a leading cause of morbidity and mortality in healthcare, with an annual loss of billions of dollars^8–10^. Several computational approaches have been proposed for predicting side effects of a given drug^11–16^. Yet, the application of these methods in drug risk–benefit assessment is limited, as they can only predict the presence or absence of a drug side effect, not its frequency.

While the accurate estimation of the frequencies of side effects is vital to patient care in the clinical practice, it is also essential for pharmaceutical companies as it reduces the risk of drug withdrawal from the market^17,18^ or of a costly reassessment of side effect frequencies through new clinical trials^19^.

Here we present a machine learning approach for predicting the frequencies of drug side effects. We show the usefulness of our approach for drugs from multiple therapeutic classes and side effects belonging to all physiological systems. Given a small number of experimentally determined side effects, our method predicts the frequencies of a broader range of unknown side effects. To our knowledge, this is the first computational method that successfully addresses the problem of predicting the frequencies of drug side effects. A critical application of our approach is in the early phase of clinical trials, where computational predictions can be used as complementary hypotheses to set the direction of the risk assessment in later phases of clinical trials. Our approach for predicting the frequencies of drug side effects is to use a matrix decomposition algorithm that learns a small set of latent features (or signatures) that encode the biological interplay between drugs and side effects. Our model is inspired by movie recommendation systems^20–22^ that recommend movies to users: our recommendation system recommends side effects to drugs. Importantly, we constrain our matrix decomposition to be non-negative; this has the advantage of making explicit the parts-based representation^23^ thus offering biological interpretability. In other words, drugs are characterised by a set of learned non-negative features that, when additively combined, account for the side effect frequencies across the entire repertoire of drugs. Consequently, our predictions are explainable, and the individual features can be interpreted in terms of drug effects on specific human physiological systems. Here we also show that these features are related to different routes of administration and that they capture shared drug clinical activity, drug targets and anatomy/physiology of side effect phenotypes.

## Results

### The matrix decomposition model

We used the Side effect Resource (SIDER) 4.1 database^24^ to obtain the frequencies of drug side effects and analysed drugs with known Anatomical, Therapeutic and Chemical (ATC) code (see Methods). Following common practice in clinical trials^25^, we used five frequency classes to quantify the occurrence of side effects—the standardisation of frequency formats is explained in Supplementary Note 1 (Supplementary Fig. 1). By coding side effect frequency classes with integers between 1 and 5 (Supplementary Table 1)—very rare = 1, rare = 2, infrequent = 3, frequent = 4, very frequent = 5—we assembled an *n* × *m* matrix *R*, containing 37,441 frequency class associations for *n* = 759 drugs and *m* = 994 unique side effects (Supplementary Data 1). The remaining entries in *R* were filled with zeros.

**Fig. 1:**
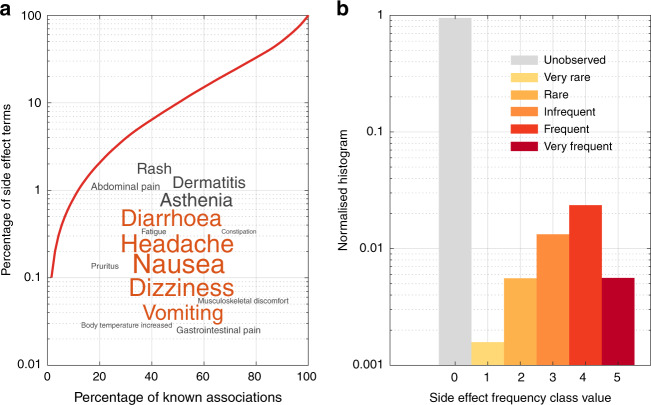
Distribution of drug side effects in our data set. **a** Long-tailed distribution of side effects. Side effects in *y*-axis are ordered in decreasing order of popularity, i.e. the number of drugs in which a side effect appear. Inset. Word cloud of the 15 most popular side effects. The size of the word is proportional to its popularity; the five most popular ones are coloured in orange. **b** Histogram of side effect frequency classes. The frequency of a drug side effect in the population can be very rare (<1 in 10,000), rare (1 in 10,000 to 1 in 1000), infrequent (1 in 1000 to 1 in 100), frequent (1 in 100 to 1 in 10) or very frequent (>1 in 10)—shown in shaded red bars. The remaining of the associations are unobserved (grey bar).

The average frequency value in *R* is 3.52, indicating that frequencies from clinical trials are biased towards frequent side effects. This has been attributed to the limitation of clinical trials at detecting side effects of rare occurrence^26^. Popular side effects, such as headache, account for most of the non-zero entries in *R*, indicating that there are specific side effects that are reported on most drugs. Indeed, our analysis of *R* showed that drug side effects follow a long-tailed distribution (Supplementary Fig. 2), where about 30% of the side effects are responsible for 80% of the associations (Fig. 1a). Figure 1b shows that the distribution of frequency classes in *R* is zero-inflated, meaning that about 95% of the associations are unobserved.

The long-tailed distribution of side effects resembles the distribution of the ratings previously found in movie datasets, such as Netflix or Movielens^27^ (Supplementary Fig. 3). One widely studied group of methods for movie recommendation systems is based on matrix decomposition techniques^28^. Their fundamental assumption is that both users and movies can be represented as latent feature vectors in a low-dimensional space and that a rating value for a specific user–movie pair is obtained by the dot product of the corresponding feature vectors. The assumption is reasonable for movie datasets, where latent features can be thought of as modelling both movie genres and user preferences (e.g. thriller, romance, sci-fi).

We realised that this assumption is also reasonable for our task: drugs and side effects can be represented as latent feature vectors in a low-dimensional space where the latent features might capture specific molecular or cellular mechanisms that elicit side effects^29^. Therefore, our idea is to learn a low-dimensional latent representation for each drug—that we shall call drug signature, ${\mathbf{w}} \in {\Bbb R}^k$—and a low-dimensional representation for each side effect—side effect signature, ${\mathbf{h}} \in {\Bbb R}^k$—such that the frequency of a drug–side effect pair is obtained by the dot product of the two feature vectors. This amounts to decomposing *R* into a product of two matrices as *R* ≈ *WH*, where $W \in {\Bbb R}^{n \times k}$ (each row is a drug signature), $H \in {\Bbb R}^{k \times m}$ (each column is a side effect signature) and *k* << min(*n*, *m*) is the number of latent features in our model (see Methods). Our matrix decomposition algorithm learns the matrices *W* and *H* by minimising the following loss function:

1 $$\mathop {{\min }}\limits_{W,H} {\cal{L}}(W,H) = \frac{1}{2}\mathop {\sum }\limits_{\{ \left( {i,j} \right)|R_{ij} \in {\mathrm{{\Omega} }}\} } (R_{ij} - (WH)_{ij})^2 + \frac{\alpha }{2}\mathop {\sum }\limits_{\{ \left( {i,j} \right)|R_{ij} \in \{ 0\} \} } \left( {WH} \right)_{ij}^2$$

subject to the non-negative constraints *W*, *H* ≥ 0.

The first summation in our model is the fitting constraint on the observed entries, which aims at reconstructing *R* for the known frequency classes in ${\mathrm{{\Omega} }} \in \left\{ {1,2,3,4,5} \right\}$. This term is commonly used in collaborative filtering models to learn from users’ ratings on movies. The second term in Eq. (1) is the fitting constraint on the zeros, which aims at reconstructing the zeros found in *R*, and we introduced it here because our data set is fundamentally different from movie ratings. While in the movie rating matrix a zero entry is simply a missing value that needs to be filled in, for our problem, a zero entry indicates that a specific side effect was not detected for a given drug—which could either mean that the drug does not cause the side effect or that it does, but it could not be detected. The parameter $\alpha \in [0,1]$ controls the relative importance of the zeros during learning; in other words, it represents our confidence in their correctness. Observe that the second term also acts as a regularisation factor, so no additional regularisation term is required (Supplementary Note 2). Finally, we impose non-negative constraints on our solution as it favours biological interpretability since only additive combinations of the latent features are allowed^30^.

**Fig. 2:**
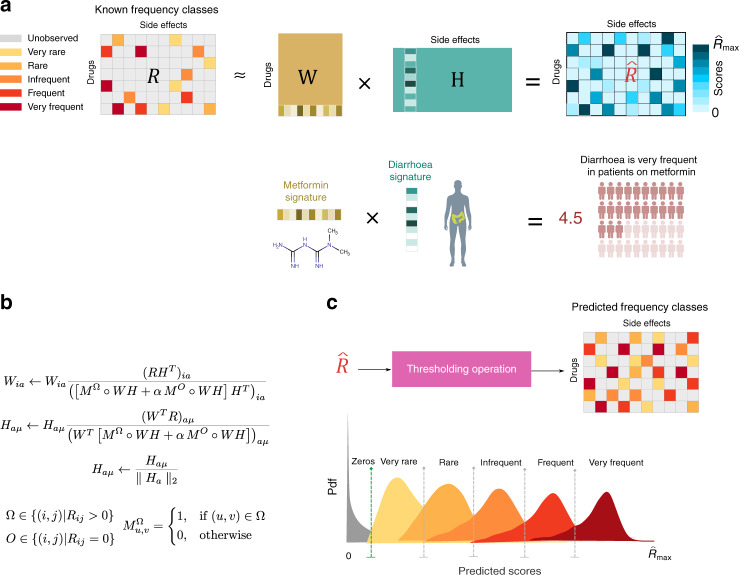
Overview of our approach. **a** Thirty-seven thousand four hundred and forty-one known frequency associations for 759 therapeutically diverse drugs and 994 side effects from all human physiological systems were collected from the SIDER 4.1 database. The associations were standardised into the five frequency classes commonly used in randomised controlled trials and arranged into an *n* × *m* matrix *R* by encoding them using integers: very rare (=1), rare (=2), infrequent (=3), frequent (=4) and very frequent(=5). Unobserved associations were encoded with zeros. Our algorithm decomposes the matrix *R* into the product of two matrices, *W* (of size *n* × *k*) and *H* (of size *k* × *m*). By multiplying the matrices *W* and *H*, we obtain $\hat R$, which models *R*, and where all the integers are replaced by real numbers—these are our predicted scores. Note that values that replace the zeros in *R* will constitute our predictions. Rows of *W* are the drug feature vectors (drug signature); columns of *H* are the side effect feature vectors (side effect signature). The lower illustration depicts how our model discovers a low-dimensional signature vector for the anti-diabetic drug Metformin and a low-dimensional signature vector for the side effect diarrhoea, such that the dot product of these two signatures models the frequency of diarrhoea in patients on Metformin. The body parts infographic vector was created by macrovector—www.freepik.com. **b** Starting from non-negative initial conditions for *W* and *H*, iterations of these update rules find *W* and *H*, an approximate factorisation for *R* by converging to a local minimum of the objective function in Eq. (1). **c** Frequency classes were obtained from $\hat R$ using a thresholding operation. Thresholds are set by maximum likelihood using likelihood density functions for each class that are estimated during cross-validation.

An overview of our approach for predicting the frequencies of drug side effects is presented in Fig. 2 (and Supplementary Notes 4 and 5). The starting point is the matrix *R* containing an encoding of the side effect frequency classes for each drug (Fig. 2a). We learn the matrices *W* and *H* that minimise the loss function in Eq. (1), by employing an iterative algorithm that uses a simple multiplicative update rule (Fig. 2b, see Methods). Our algorithm, inspired by the diagonally rescaled principle of non-negative matrix factorisation^30^, is fast, it does not require setting a learning rate nor applying a projection function and it satisfies the Karush–Kuhn–Tucker (KKT) complementary conditions of convergence (proof in Supplementary Note 3). Having learned *W* and *H* such that $R \approx WH$, we calculate the matrix $\hat R = WH$. Notice that, while *R* contains integers in the range [0, .., 5] that are our original data, $\hat R$ contains real positive numbers that are our predicted scores. Finally, to assign specific frequency classes to the predicted scores in $\hat R$, we apply a thresholding operation (Fig. 2c). The methodological details are given in the next sections.

### Predicting frequencies of side effects for multiple drugs

We started by analysing the performance of our method at recovering missing associations in the matrix *R*. To do this, we held-out 10% of the observed associations in our data matrix *R* for testing. The remaining 90% were used in a tenfold cross-validation procedure to set the two parameters of our algorithm: *k* (the number of latent features) and *α* (confidence in the zeros)—see Methods. During cross-validation, we assessed the prediction performance using both the root mean squared error (RMSE) on the frequency classes and the area under the receiver-operating curve (AUROC) obtained when predicting the presence/absence of the associations (binary classification problem)—the latter has been previously used by Cami et al.^11^ for the same problem. Here the first metric measures distance from the correct frequency class values while the second one guarantees the detection of correct associations. We obtained an excellent performance with *α* = 0.05 and *k* = 10; mean and s.t.d. RMSE = 1.372 ± 0.021, and mean and s.t.d. AUROC = 0.920 ± 0.003 (Supplementary Fig. 4). The performance of the algorithm is robust with respect to the setting of the parameters *α* and *k* (Supplementary Figs. 5 and 6). Given that our data set is highly imbalanced, we also analysed the binary classification performance of our method using the area under the precision-recall curve (AUPRC). Following the procedure in Luo et al.^31^, we calculated the AUPRC when the ratio between the binary classes in the test set varies from 1 to 10. Supplementary Fig. 7 shows that the mean AUPRC varies from 0.914 ± 0.003 to 0.594 ± 0.0084.

**Fig. 3:**
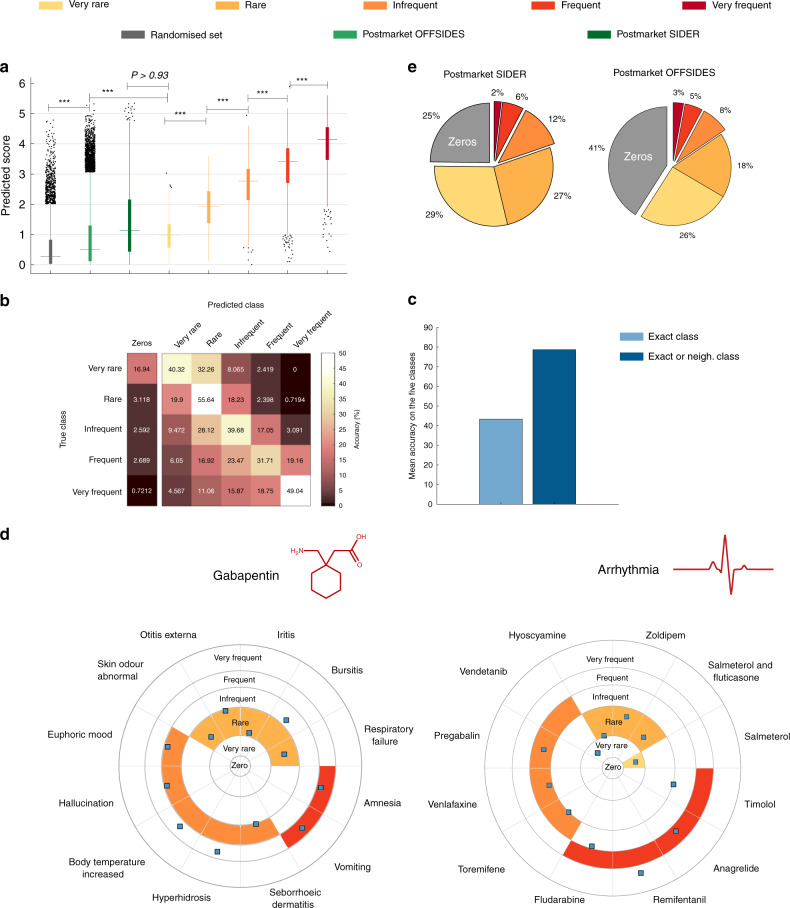
Evaluation of our method on multiple drugs. **a** Box plots of the predicted scores obtained for the three test sets: (1) predicted scores for each of the five frequency classes in the held-out test set (in yellow to red scale). The differences in the distributions are statistically significant: rare vs very rare (*P* < 2.80 × 10^−12^), infrequent vs rare (*P* < 1.31 × 10^−40^), frequent vs infrequent (*P* < 3.45 × 10^−51^) and very frequent vs frequent (*P* < 9.00 × 10^−26^); (2) SIDER and OFFSIDES post-marketing (in green scale); (3) randomised post-marketing set (in grey). The differences in the distributions between the randomised set and the other sets are statistically significant (largest *P* < 1.80 × 10^−20^). Significance levels between the scores are indicated with asterisks (***P ≤ 0.001). One-tailed Wilcoxon rank-sum test was used in all the cases. **b** Accuracy percentages for the predictions in the held-out test set. **c** Mean accuracy on the exact class together with the mean accuracy at predicting the exact class or one of the neighbouring classes. **d** Illustrative examples from the held-out test set. Twelve randomly chosen predictions for the anticonvulsant drug Gabapentin (left) and the cardiovascular side effect arrhythmia (right) are shown around polar plots, each in a dedicated sector. Grey concentric circles between frequency classes correspond to thresholds learned by maximum likelihood. The correct class for each association is coloured in each circular sector, while predicted scores are shown as blue squares. **e** Pie chart of predicted frequency classes assigned to the SIDER and OFFSIDES post-marketing test sets.

On the held-out test set, our model scored an RMSE of 1.32 and an AUROC of 0.932 (and an AUPRC of 0.59 for a class imbalance ratio of 10; PR curves shown in Supplementary Fig. 8). Figure 3a shows, for each of the five frequency classes in the test set, the box plots of the distribution of the values that were predicted for that class (Supplementary Fig. 9). The Pearson correlation between the predicted scores and their corresponding frequency classes was *ρ* = 0.47 (significance, *P* < 2.40 × 10^−209^). The differences between the distributions of scores for the five frequency classes were statistically significant (Kruskal–Wallis one-way analysis of variance significance at 1%, *P* < 1.15 × 10^−193^).

To predict the specific frequency class of a given association, we need a way to assign predicted scores to frequency classes. Due to incomplete data and biases on the observed entries, we cannot obtain reasonable estimates for the priors for each class. Therefore, we assigned scores to classes based on maximum likelihood using the distributions obtained from the validation sets during cross-validation (see Fig. 2c, see Methods). Furthermore, due to the lack of experimentally validated zero values, in order to discriminate the zero associations, we followed an approach similar to the one used by Cami et al.^11^ and chose a threshold using the ROC curve at a sensitivity of 0.97 given a specificity of 0.57.

Figure 3b, c shows the accuracy at predicting side effect frequency classes on the held-out test set. For any given class, the most predicted class is the correct one, and the prediction accuracy ranges from 55.2 to 75.5% when including the contiguous lower class and 67.8 to 94% when both contiguous classes are considered. Looking at the first column in the figure, we notice how our system rarely (0.72%) fails to detect a very frequent side effect and seldom misses side effects in the frequent (2.68%), infrequent (2.52%) and rare (3.11%) classes. The number of undetected side effects only increases for the very rare class (16.94%) (Supplementary Fig. 10), probably due to the small number of known associations in this class. As illustrative examples, Fig. 3d (and Supplementary Figs. 11 and 12) presents the predicted scores and the exact frequency classes for the side effects of the anticonvulsant drug Gabapentin, a top-50 prescribed drug in the U.S.^32^, and for the side effect arrhythmia, critical in cardiotoxicity assessment^33^.

We also tested the accuracy of our method when the percentage of associations that were removed (and predicted back) from *R* varied from 10 to 70%. We found that the mean accuracy of our method is very robust when increasing amounts of data are randomly missing from the matrix *R* (Supplementary Fig. 13).

We further tested the performance of our system at predicting the frequencies of side effects that were discovered after the drug entered the market (post-marketing side effects, hereafter). This amounts to a prospective evaluation where post-marketing data appear only in the test set—it is a realistic scenario that preserves the chronological order in which the information becomes available^11^. We collected two independent post-marketing test sets: (i) the post-market SIDER^24^ test set containing 9387 post-marketing associations; and (ii) the post-market OFFSIDES^34^ test set containing 36,032 associations (see Methods; Supplementary Data 2). The SIDER test set corresponds to post-marketing side effects reported in drug leaflets, whereas the OFFSIDES test set corresponds to statistically significant side effects reported in the Adverse Event Reporting System (AERS)^35^. Arguably, the associations provided in the SIDER database extracted from drug leaflets are more reliable because they are curated by pharmacological experts. Importantly, both post-marketing sets only inform us about the presence or absence of the side effects, not its frequency. Yet, post-marketing side effects are typically regarded as side effects of very rare occurrence in the population^26,34^ because they are not detected in clinical trials.

Strikingly, the statistical analysis of the distribution of scores predicted for the SIDER post-market test set showed no significant differences from the scores predicted for the very rare class in the held-out test set (Fig. 3a, one-tailed Wilcoxon sum-rank significance, *P* > 0.936), indicating that our method predicted, on average, very rare scores for these post-marketing side effects despite the small number of very rare associations in *R* (only 3.2% are very rare). This was not the case for the OFFSIDES test set, which ranked significantly lower (Fig. 3a, one-tailed Wilcoxon sum-rank significance, *P* < 6.25 × 10^−8^). Figure 3e shows that a large percentage of the post-marketing associations in both test sets were predicted either as rare or very rare (44–55%).

To check whether the predicted scores for the post-market test sets was simply due to random chance, we selected a randomised test set of the same size of the SIDER post-market test set, by sampling entries from the $\hat R$ matrix corresponding to zero-value entries in the matrix *R* that had been used for training. We found that the distribution of scores predicted for this randomised test set was significantly lower than any of the post-marketing test sets (OFFSIDES test set vs randomised set, one-tailed Wilcoxon sum-rank significance, *P* < 1.45 × 10^−177^; SIDER post-marketing vs randomised set, *P* < 2.23 × 10^−308^). This means that many of these very rare side effects, which were discovered from post-market observational databases, could have been systematically predicted by our approach.

### Predicting frequencies of side effects for single drugs

**Fig. 4:**
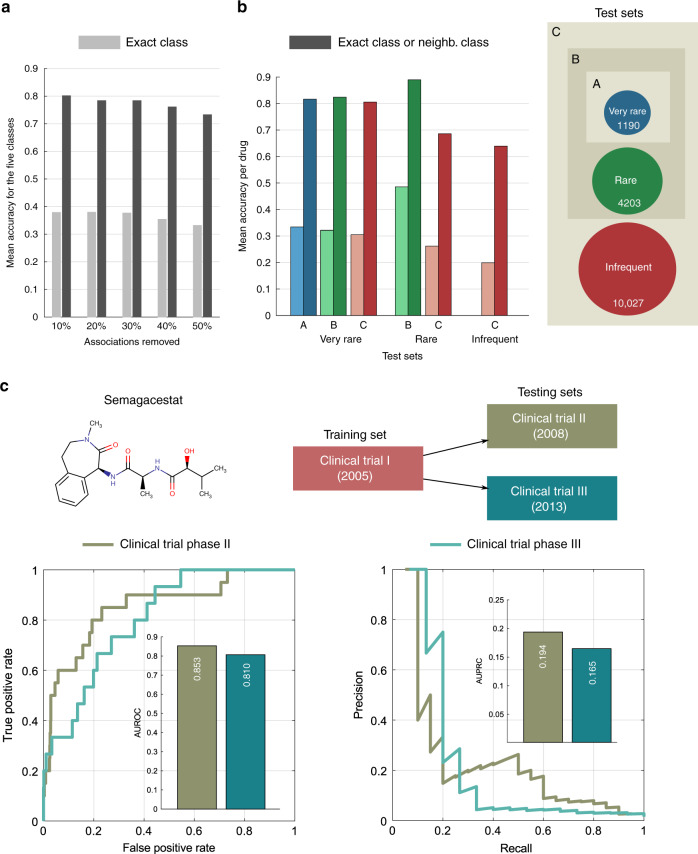
Evaluation of our method on single drugs. **a** Mean accuracy at predicting the exact class (light grey) and the exact class or one of the neighbouring classes (dark grey) for varying percentages of randomly chosen associations. **b** Mean accuracy at predicting the exact class (light colour) and the exact class or one of the neighbouring classes (dark colour) for test sets A, B and C. Set A (blue bars) = 1190 very rare associations, 126 drugs; set B (green bars) = set A + 4203 rare associations, 237 drugs; set C (red bars) = set B + 10,027 infrequent associations, 473 drugs. Each test set is represented with circles, circle size represents the number of associations in each test set. **c** A case study for the γ-secretase inhibitor Semagacestat, an investigational small molecule for the causal treatment of Alzheimer’s disease. Side effect frequencies from three chronologically different phases of randomised controlled trials were collected: phase I (2005), phase II (2008) and phase III (2013). Only 8 (2 frequent and 6 very frequent) associations from phase I were used to train the model and the remaining associations of phases II (13 frequent and 7 very frequent), and III (13 frequent and 2 very frequent) were used separately to test the model. Scores generated by the model for the 986 unobserved side effects were assessed against the known associations from phases II and III using the receiver-operating characteristic curve and precision-recall curves (inset bar plots of AUROC and AUPRC).

A key question for the real applicability of our approach concerns its ability to predict the frequency of side effects for single drugs. In practice, for a given drug, frequencies of side effects are discovered incrementally in a specific chronological order during phases I, II and III of clinical trials. We realised that we could simulate this incremental discovery process by randomly removing (and predicting back) an increasing percentage of associations for a single drug (see Methods). Figure 4a shows the mean accuracy at predicting the exact class together with the accuracy at predicting the exact class or one of the neighbouring classes. The figure shows that our method’s accuracy is robust even when a high percentage of associations for a given drug is missing—the mean accuracy only drops by 4.69% (exact class) when 50% of the associations for each drug are removed. Notice also that the results when removing 10% of the associations from each drug are not very different from our earlier results (in Fig. 3b), obtained by removing randomly 10% of associations from the entire matrix *R*.

During clinical trials, typically, more frequent side effects are discovered first, in earlier phases of clinical trials, while more rare side effects are discovered in later phases. We performed a more realistic simulation that takes this chronology into account where, for each drug, we removed side effects belonging to an increasing number of classes. First, we removed only the very rare side effects (test set A in Fig. 4b), then we repeated the procedure by removing together very rare and rare associations (test set B in Fig. 4b) and finally we repeated the procedure by removing together very rare, rare and infrequent side effects (test set C in Fig. 4b)—see ‘Methods’. Figure 4b shows the mean accuracy of our method at predicting the exact frequency class and the accuracy at predicting the exact class or one of the neighbouring classes. The figure shows that our method can accurately predict more rare side effects for single drugs even when only more frequent side effects are available. These results suggest the potential usefulness of our method for predicting frequencies of side effects using data from early phases of clinical trials.

To further prove this concept in a real case study, we used the outcomes of clinical trials for the γ-secretase inhibitor Semagacestat, the only drug for which we could find detailed information on the outcome of each phase of clinical trials. We manually curated the data on the frequencies of side effects from randomised controlled trial phases I^36^, II^37^ and III^38^, conducted for Semagacestat in 2005, 2008 and 2013, respectively (Fig. 4c and Supplementary Data 3). Only a small number of relevant frequent and very frequent side effects were reported in detail for each clinical study, and after filtering placebo side effects of higher frequency, the number of side effect associations in phases I, II and III were 8, 25 and 15, respectively. We then added Semagacestat side effects from phase I to our data matrix *R* by adding a new row in the matrix (Supplementary Fig. 14 and Supplementary Note 4) and then trained our method using all the available data (with *α* = 0.05 and *k* = 10). After ranking all the 986 unobserved side effects for Semagacestat, we measured the performance of our method at recovering the new frequent side effects that were observed in phases II and III, obtaining an AUROC of 0.853 and 0.810, respectively—see Fig. 4c for the ROC and PR curves. This indicates that our method would have been able to suggest many of these side effects already after the results of clinical trial phase I.

The small number of side effects available for Semagacestat in phase I of clinical trials prompted us to ask how much the accuracy of our method for a given drug depends on the total number of side effect associations already known for that drug. To assess this accurately, we used a leave-one-out procedure, in which for each association in our data matrix: (i) we removed it from the matrix; (ii) we trained our model with the remaining associations; (iii) we predicted the frequency class for that missing association. The number of known side effects for the different drugs varies greatly, and thus this experiment allowed us to analyse the dependency between accuracy and number of side effects. We observed that the accuracy of our method is very robust to variations in the number of side effects available for each drug. Our method predicts accurately the frequency of the side effects even for drugs with only 8–10 known side effect frequencies (Supplementary Fig. 15).

Finally, we checked whether the chemical similarity between drugs was somehow affecting our evaluations of the method’s accuracy. To do this, we measured the performance of our method for sets of drugs with different range of chemical similarities. We performed this analysis on 754 drugs in our data set. For each drug, we tested the performance of our method at predicting 10% of the associations that were held out in a test set, while varying the level of chemical similarity with other drugs in the training data sets (the percentage of drugs that are kept for training for distinct thresholds of chemical similarity is shown in Supplementary Fig. 16). Supplementary Fig. 17 shows that the performance of our method is very robust with respect to the amount of chemically similar drugs present in the training data set. The values for the similarity thresholds used in these experiments were motivated by a comparative analysis of the chemical diversity of our data set performed using compounds from the Drug Repositioning Hub^39^ (see Supplementary Note 6 and Supplementary Fig. 18).

### Drug signatures are informative of main drug activity

The effectiveness of our model at predicting the frequency of side effects prompted us to analyse whether the learned signatures are informative of the biology underlying drug activity.

We began by analysing whether the signatures were reproducible across independent runs. Using all the available data in *R*, we followed the reproducibility procedure used by Alexandrov et al. to study cancer mutational signatures^40,41^ (see Methods). We found that eight out of the ten components of the signatures have a median reproducibility score >80% (Supplementary Figs. 19 and 20). Hereafter, we report the results found for the best solution over multiple runs (Supplementary Data 9 and 10).

**Fig. 5:**
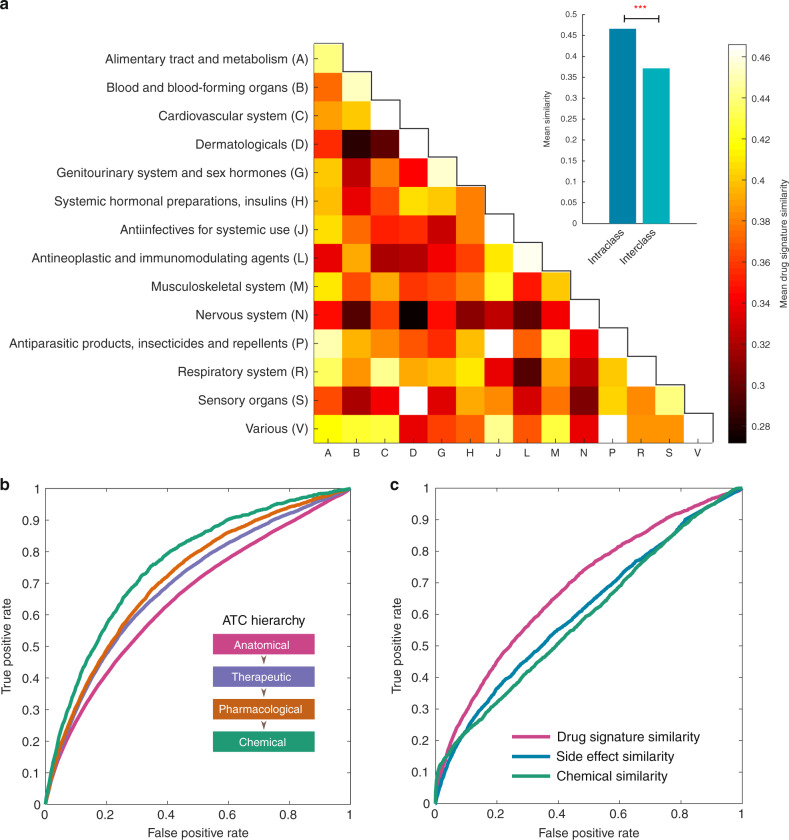
Drug signatures capture drug clinical and molecular activity. **a** Heat maps of mean drug signature similarities per anatomical class. Each (*x*, *y*) tile represents, for each main Anatomical, Therapeutic and Chemical (ATC) drug category, the mean similarity of drug pairs where one drug belongs to category *x* and the other to category *y*. The value ranges from 0.27 (Nervous system—dermatological) to 0.55 (Nervous system—nervous system). The colours range between the minimum mean similarity and 0.466, with all values >0.466 (in the diagonal: 0.471 (C), 0.512 (D), 0.55 (N), 0.47 (P), 0.52 (R), 0.475 (V)) set to 0.466. Inset: the average intraclass similarity is significantly higher than the average inter-class similarity (*t* test significance, *P* < 2.62 × 10^−13^. **b** ROC curve representing the ability of the drug signature similarity to discriminate pairs of drugs that share Anatomical, Therapeutic and Chemical (ATC) category at each of the different levels in the ATC taxonomy. Drug signature similarity was predictive of clinical drug activity at different levels: anatomical class (38,711 pairs share vs 248,950, AUROC = 65.33%), therapeutic subclass (11,960 pairs share vs 275,701, AUROC = 69.51%), pharmacological subclass (5,522 pairs share vs 282,139, AUROC = 71.54%) and chemical subclass (1,736 pairs share vs 285,925 do not, AUROC = 76.05%). **c** ROC curve representing the ability of the drug signature similarity, side effect similarity and Tanimoto chemical similarity scores to discriminate pairs of drugs that share targets. For 435 drugs in our data set, 2,808 pairs were known to share molecular targets, whereas 91,587 pairs were unknown.

Having shown that the features were highly reproducible allowed us to investigate the link between drug signatures and drug clinical activities. We hypothesised that the signature for two drugs should be similar when they share clinical activity. Clinical activity for drugs was defined based on their main ATC class level (Supplementary Table 2 and Supplementary Data 4)—a five-level hierarchical organisation of terms where lower levels of the hierarchy contain more specific descriptors of clinical activity. Figure 5a shows that the similarity between the signature of drugs within an ATC class is higher than the similarity between classes (Supplementary Fig. 21a, see Methods).

We then formulated a simple binary classification task in which the signature similarity was used to predict drugs that shared the same clinical activity at each level of the ATC hierarchy (see Methods). We observed that the prediction performance increases when considering terms located lower in the ATC hierarchy (Fig. 5b and Supplementary Fig. 21b). Our findings correctly reflect the fact that drug clinical activity becomes more similar as we move to lower (more specific) levels of the ATC hierarchy.

In a similar way, we checked whether the drug signature for two drugs is more similar when they share drug targets (see Methods, Supplementary Fig. 22). We found that drug signature similarities are predictive of shared protein targets between drugs (AUROC = 68.38%; Fig. 5c). Interestingly, the predictions are better than baselines previously used elsewhere^34,42^, such as the two-dimensional (2D) Tanimoto chemical similarity (AUROC = 59.26%) and the Jaccard side effect similarity (AUROC = 61.07%).

We also analysed side effect signatures attempting to reveal a link between these signatures and the anatomy/physiology of the side effect phenotypes. Side effects were grouped based on their system organ classes according to the Medical Dictionary for Regulatory Activities (MedDRA) terminology (Supplementary Table 3 and Supplementary Data 5 and 6). We found that signatures for two side effects tend to be more similar when they are phenotypically related (Supplementary Fig. 23). Moreover, the similarity between side effect signatures is predictive of shared MedDRA category at each of the different levels of the MedDRA hierarchy, and predictions improve as we move to more specific terms in the hierarchy (Supplementary Fig. 24).

It is important to note that in these analyses we formulated binary classification problems merely to assess whether drug signatures (or side effect signatures) are informative of the biology underlying drug activity. We are not introducing here new methods for predicting drug targets or drug indications. These experiments show that there is a relation between similarity between drug signatures and similarity between drug activities at the anatomical and molecular level—the formulation of binary classification problems has been used before to verify a relationship between a specific feature and a property under study^34,42,43^.

### Interpreting the meaning of the signature components

We have shown that the signatures of drugs and side effects, as a whole, encode meaningful biological information of drug molecular and clinical activity. A further important question is whether the individual components of the signatures are interpretable, i.e. have any biological or pharmacological interpretation.

We grouped drugs and side effects according to their main anatomical classes, and we looked for significant activations of individual components of the signatures for each group. The groups were obtained using top-level terms in ATC and MedDRA hierarchies, respectively. Significant relationships are provided in Supplementary Data 11–16. Strikingly, we observed that, often, specific components of the signatures were significantly activated for drugs and side effects that were anatomically related—Table 1 summarises the correspondences that we found to be statistically significant (one-tailed Wilcoxon rank-sum test with Benjamini–Hochberg adjusted significance, *P* < 0.01).

**Table 1:** Statistically significant associations with each signature component.

Signature component	Anatomical drug ATC category	Physiological side effect MedDRA category	Comments
1	Genitourinary and sex hormones (G), antineoplastic and immunomodulating agents (L)	Reproductive system and breast, musculoskeletal and connective tissues	Strongly associated with endocrine therapy drugs (L02) and sex hormones and modulators of the genital system (G03) drugs. Weakly with N06 (psychoanalytic)
2	Cardiovascular (C)	Cardiac, vascular and respiratory, thoracic and mediastinal	Also associated with anaesthetics (N01). Strongly associated with arrhythmias
3	Nervous system (N)	Respiratory, thoracic and mediastinal	A weak, less stable signature. Associated with antimycotics (J02) and psychoanalytic (N06)
4	Dermatological (D), sensory organs (S)	Skin and subcutaneous tissue, eye and immune system	Strongly related to epidermal and dermal conditions, ocular infections, irritations and inflammations, including allergic conditions. Also associated with the nasal and transdermal delivery administration
5	Nervous system (N)	Nervous system, psychiatric disorders	Specific to nervous system drugs. It is associated with many subcategories of nervous system drugs, except anaesthetics (N01). Also, only weakly associated with psycholeptics (N05). Equal neurologic and psychiatric side effects
6	Respiratory system (R)	Respiratory, thoracic and mediastinal, infections and infestations	Also associated with drugs used in diabetes (A01), lipid-modifying agents (C10) and urological (G04), highlighting some interactions with metabolism/haemostasis. Also associated with inhalation and nasal administration
7	Anti-infectives for systemic use (J)	Gastrointestinal	Also linked to drugs for acid-related disorders (A02)
8	Nervous system (N)	Nervous system disorders, psychiatric disorders	Specific to nervous system drugs. Specifically, antipsychotics and anxiolytics (N05A/B). More psychiatric side effects. Prominently associated with mood and sleep disorders and disturbance. Associated with oral administration
9	Antineoplastic and immunomodulating agents (L), anti-infective for systemic use (J)	Metabolism and nutrition, investigations, blood and lymphatic system	It is associated with antineoplastic agents (L01), antimycotics and antivirals, both for systemic use (J02/05). Also, with immunosuppressant drugs (L04). Associated with electrolyte and fluid balance conditions and hepatobiliary investigations
10	Antineoplastic and immunomodulating agents (L)	Blood and lymphatic system, vascular disorders	Strongly associated with antineoplastic agents (L01) and weakly with antithrombotic (B01). Associated with haemorrhagic vascular disorders

We investigated some entries of the table in detail. Component 1 of the signature is significantly associated with the sex hormone drugs and with the breast disorder-related side effects (adjusted significance, *P* < 4.01 × 10^−12^). When we performed a more in-depth pharmacological analysis by looking at lower levels in the ATC hierarchy (finer granularity), our analysis revealed that this correlation mostly comes from sex hormones and modulators of the genital system (G03) drugs and endocrine therapy (L02) drugs (adjusted, *P* < 4.06 × 10^−6^ and *P* < 1.24 × 10^−9^, respectively).

Another notable example is component 8 of the signatures. This component is specific to neurological drugs (adjusted, *P* < 3.48 × 10^−31^, but *P* > 0.05 for all other drug classes) and to side effects related to the nervous system and psychiatric disorders. In-depth pharmacological analysis reveals that this component is linked to antipsychotics and anxiolytics drugs and with psychiatric side effects (mood and sleep disorders). Conversely, component 5 of the signatures, which is also specific to neurological drugs (adjusted, *P* < 1.82 × 10^−12^, *P* > 0.05 for all other drug classes), has more balanced neurological and psychiatric side effect profiles.

In some cases, the signature components can be associated with more than one anatomical class, and the connection between the classes becomes only apparent after considering the off-target or off-tissue effect of the drugs. As an example, consider component 2 of the signatures, which is strongly associated with both cardiovascular system drugs (adjusted, *P* < 7.13 × 10^−10^) and cardiac and vascular-related side effects (adjusted, *P* < 5.24 × 10^−4^ and *P* < 2.56 × 10^−17^, respectively). There is, however, an unexpected link with nervous system drugs. In-depth pharmacological analysis reveals that component 2 is linked to anaesthetic drugs (N01)—the only neurological drugs associated with this component (adjusted, *P* < 1.25 × 10^−2^). Conversely, anaesthetic drugs are not statistically significantly associated with any other components—including components 5 and 8, which are neurological specific, as we described above. Anaesthetic drugs reportedly affect the regular cardiac electrical activity by interacting with the ion channels—the component 2 of the signatures is indeed strongly associated with arrhythmias (adjusted, *P* < 8.88 × 10^−10^).

Furthermore, it is well known that drug route of administration affects the side effects. We tested whether components in the signatures can capture this relation (data provided in Supplementary Data 7). We found that specific components of the drug signatures are significantly associated with several routes (Supplementary Data 17). Component 6 of the signatures—associated with the respiratory system—is associated with inhalation and nasal administration. Component 4—associated with the dermatological system—is also associated with nasal administration and transdermal delivery administration, which is typically known to cause adverse skin reactions. Finally, we found that component 8—associated with the nervous system—was associated with oral administration. We note, however, that this last association could be due to a large number of nervous system drugs in our data set (see Supplementary Table 2).

## Discussion

The correct identification of frequencies of drug side effects is critical to avoid clinical trial failures^44^ or the withdrawal of drugs from the market. In this paper, we introduced an interpretable machine learning approach to predict the frequencies of unknown side effects for drugs with a small number of determined side effect frequencies. Our model learns a low-dimensional representation of drugs and side effects that we called signatures. We showed that these signatures encode meaningful biological information about drug activity at the anatomical and molecular level.

We envision the use of our system by safety professionals working in pre- and post-marketing drug development: in the premarketing phase, to assist in the design of clinical trials by generating a hypothesis on the frequencies of certain side effects; in the post-marketing phase, to complement the evidence from observational databases for the early discovery of side effects of very rare occurrence—this requires an analysis of the low scores predicted by our system (Supplementary Note 5). The interpretability of our model can also assist policymakers and regulatory agencies when assessing the safety of candidate compounds or of drugs that are already in the market. To assist in this task, we provide the complete set of predictions by our model in Supplementary Data 8.

To the best of our knowledge, this is the first method that can predict the frequencies of drug side effects in the population. Earlier methods can predict the probability of a given drug side effect association, but these probabilities are only weakly correlated with the side effect frequencies and therefore they cannot be used effectively for the prediction of frequency classes—we verified this for the scores obtained by the predictive pharmaco-safety networks (PPN-NET)^11^ (Supplementary Fig. 25).

An innovative technical aspect of our matrix decomposition algorithm is that it can take into account different levels of uncertainty associated with the data. The underlying assumption of our model is that the matrix is fully—rather than partially—observed but that a well-defined set of entries are noisy—in our problem, these are the zeros, corresponding to unobserved drug side effect associations. Earlier matrix decomposition methods, such as singular value decomposition or non-negative matrix factorisation (NMF)^30^, do not explicitly account for different levels of uncertainty in the data. Our multiplicative learning rule is simple, computationally efficient and has theoretical guarantees of convergence. We envisage its use for other problems in which there is prior knowledge on the uncertainties associated with distinct subsets of associations in the data (see our detailed discussion in Supplementary Note 2)—for example, for the problems of predicting protein–RNA interaction^45^ and disease gene prediction^46^, as well as in social network analysis, and recommendation systems for e-commerce.

The seminal work of Campillos et al.^29^ had shown that drug side effects are predictive of drug targets. More recently, Wang et al.^47^ had shown that drug side effects are predictive of therapeutic indications. Therefore, one interesting question was whether our model’s signatures were able to capture these biological relationships. We found that drugs with similar signatures were more likely to share a protein target and to belong to the same anatomical, therapeutic, pharmacological and chemical category.

**Fig. 6:**
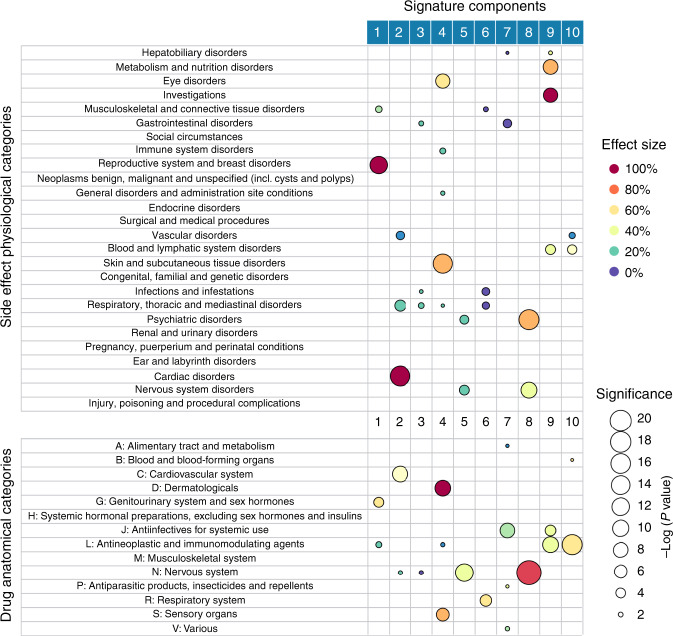
Summary of statistically significant signature activations. Drugs were grouped based on their main Anatomical, Therapeutic and Chemical (ATC) classes while side effects were grouped by their System Organ Class (SOC) categories in MedDRA. Only statistically significant associations (one-tailed Wilcoxon sum-rank test with Benjamini–Hochberg adjusted significance, *P* < 0.05) are shown. The size of the circle represents the significance (*P* value), and the colour encodes the effect size of the association—the difference between median in the group compared to the median of all drugs (or side effects).

The non-negative constraints in our model favour a part-based representation^30^ in the signatures. Thus importantly, the side effects of a given drug become explainable in terms of a combined chemical perturbation on the different parts of the human physiological system. This representation of drug activity makes sense in the context of network pharmacology^48,49^: the observed side effect patterns for a given drug can be explained by a combination of perturbations in distinct organ system networks. Figure 6 shows signature components with significant activations for anatomically related groups of drugs and physiologically related side effects. Specific components of the signatures are strongly associated with specific anatomical classes. These relationships could be useful to researchers to formulate biological hypotheses relating drugs, side effects, molecular mechanisms and human anatomical systems. The signatures could also be useful in other pharmacological research, such as in the study of frequencies of side effects produced by drug combinations.

To be used in practice, our method requires a small number of available side effects for each drug or compound. This is usually the case during clinical drug development or after the drug has entered the market. It would be important to extend our approach to predict the frequencies of side effects from compound features directly (e.g. chemical structure). This has already been attempted for the problem of predicting the presence or absence of drug side effects^50,51^.

There are limitations and biases in public databases of drug side effects. For instance, we observed that the reported frequencies of side effects are biased towards frequent ones (Fig. 1b). Recent studies also indicate that clinical trials are biased towards male gender and certain ethnicity groups: 86% of clinical trial cohorts were Caucasian dominated in 2014^52^. Numerous previous works also reported divergent drug responses in subjects with a different genetic background^53^. We envision extending our model to integrate additional metadata from clinical trials to tailor the prediction for gender- or ethnic-specific clinical groups.

## Methods

### Data sets

*Frequencies of drug side effects*: We used the SIDER database version 4.1^24,25^ to extract the frequencies of drug side effects. In SIDER, around 40% of the pairs have frequency information and each side effect term is mapped to the MedDRA vocabulary^54^. We used side effects that were MedDRA preferred terms. Frequencies of side effects were expressed in three different ways: exact values, range of values, and frequency classes. We standardised exact values and range of value formats into frequency classes according to the values in Supplementary Table 1 and then assigned an integer value to each class, as follows: very rare (=1), rare (=2), infrequent (=3), frequent (=4), and very frequent (=5). In total, our data set contains 37,441 frequency classes that cover 759 drugs and 994 side effects. Each drug in our data set has known monotherapy ATC code. A detailed explanation of the data processing is presented in Supplementary Note 1.

*Post-marketing side effects*: Two test sets of post-marketing side effects were collected. The first set was obtained from the SIDER 4.1^24^ database, from which we retrieved 9,387 post-market associations (labels ‘post-marketing’ in SIDER)—it corresponds to side effects reported in the post-marketing section of the drug’s leaflets. The second set was obtained from the OFFSIDES database^34^, from which we retrieved 36,032 significant associations—it corresponds to statistically significant post-marketing side effects reported in the AERS. Post-marketing side effects do not have frequency classes associated with them.

*Drug–protein target associations*: We retrieved the known drug–target interactions from DrugBank release 5.0.5 (2016-08-17)^55^. We mapped the drugs from SIDER to DrugBank using the PubChem IDs and the mapping provided in DrugBank. We retrieved molecular targets (section ‘targets’ of DrugBank) for 435 drugs in our data set. In total, 1,759 associations were found between the 435 drugs and 590 unique protein targets.

*Drug chemical fingerprints*: We retrieved the known drug SMILES notations from DrugBank release 5.0.5 (2016-08-17) and PubChem (by using the PubChem IDs provided in SIDER 4.1). For 754 drugs in our data set, we could obtain a binary fingerprint. We then computed the 2D Tanimoto chemical similarity from the fingerprints, using the Open-Source Cheminformatics (RDKit) package in python. We used an RDKit-specific fingerprint that is inspired by public descriptions of the well-known Daylight fingerprint. The RDKit-specific algorithm is based on hashing molecular subgraphs. A detailed explanation of how this is computed can be found in the RDKit book in the documentation online. To compute the chemical fingerprint of each drug, we used the default set of parameters: minimum path size: 1 bond, maximum path size: 7 bonds, fingerprint size: 2,048 bits, number of bits set per hash: 2, minimum fingerprint size: 64 bits, target on-bit density 0.0.

*ATC categories and route of administration*. ATC codes and route of administration of drugs were obtained from the ATC codes WHO 2018 release. The routes of administration are defined as follows: implant, inhalation, instillation, nasal (N), oral (O), parenteral (P), rectal (R), sublingual/buccal/oromucosal (SL), transdermal (TD), and vaginal (V).

### Matrix decomposition model

We modelled the estimation of the frequency of a drug side effect as a linear combination of drugs and side effects activation patterns over a set of latent representations, that we called signatures. To predict a side effect frequency score, each of the *k* components in the drug signature are multiplied by the corresponding component of the side effect signature, and then the products are summed together. Thus the frequency score of a side effect *j* for a given drug *i* can be expressed as a combination of *k* components as follow,

$$\hat R_{ij} = \mathop {\sum }\limits_{p = 1}^k W_{ip}H_{pj} = {\mathbf{w}}_i \cdot {\mathbf{h}}_j,$$

where *W*_*ip*_ indicates the (*i, p*) element of matrix *W* whose row *i*, **w**_*i*_, is the signature for drug *i* and *H*_*pj*_ indicates the (*p*, *j*) element of matrix *H* whose column *j*, **h**_*j*_, is the signature for side effect *j*.

### The objective function and decomposition algorithm

Let us denote our drug side effect matrix for *n* drugs and *m* side effects with the matrix $R \in {\Bbb N}^{n \times m}$, where $R_{ij} = 1$ if the drug side effect pair (*i, j*) is very rare, $R_{ij} = 2$ (rare), $R_{ij} = 3$ (infrequent), $R_{ij} = 4$ (frequent) or $R_{ij} = 5$ (very frequent) and $R_{ij} = 0$ otherwise. We denote the set of frequency classes with ${\mathrm{{\Omega} }} \in \left\{ {1,2,3,4,5} \right\}$ and the set of zero values with ${\rm{O}} \in \{ 0\}$. Our matrix decomposition model approximates the data matrix *R* by the product of two low-rank matrices, as follows:

2 $$\hat R = WH,$$

where $W \in {\Bbb R}^{n \times k}$ is the matrix of drug signatures (each row contains a drug feature vector) and $H \in {\Bbb R}^{k \times m}$ is the matrix of side effect signatures (each column contains a side effect feature vector). The rank of $\hat R$ is *k* << min(*n, m*), that is, the number of components in the signatures. To learn our model in Eq. (2), we minimise the following loss:

3 $$\mathop {{{\mathrm{minimise}}}}\limits_{W,H} {\cal{L}}\left( {W,H} \right) = \frac{1}{2}||M^{\mathrm{{\Omega} }} \circ \left( {R - WH} \right)||_F^2 + \frac{\alpha }{2}||M^{\rm{O}} \circ \left( {WH} \right)||_F^2$$

subject to non-negative constraints $W,H \ge 0,$

where $||.||_F^2$ is the Frobenius norm and $\circ$ indicates element-wise matrix multiplication, $M^{\mathrm{{\Omega} }},M^{\rm{O}} \in {\Bbb R}^{n \times m}$ are projection functions to discriminate between the observed and unobserved entries in *R*, that is, $M_{ij}^{\mathrm{{\Omega} }} = 1$ if $R_{ij} \in {\mathrm{{\Omega} }}$ or $M_{ij}^{\mathrm{{\Omega} }} = 0$ otherwise; $M_{ij}^{\rm{O}} = 1$ if $R_{ij} \in {\mathrm{O}}$ or $M_{ij}^{\rm{O}} = 0$ otherwise. Our matrix decomposition model assigns different levels of confidence associated with the data. $\alpha \in [0,1]$ is a model parameter that is set to account for the lower confidence on the unobserved associations in the matrix.

To minimise Eq. (3) subject to non-negative constraints, we developed an efficient multiplicative learning algorithm inspired by the diagonally rescaled principle of non-negative matrix factorisation^56^. The algorithm consists of iteratively applying the following multiplicative update rules:

4 $$W_{ip} \leftarrow W_{ip} \circ \frac{{\left( {RH^T} \right)_{ip}}}{{\left( {(M^{\it{{\Omega} }} \circ WH)H^T + \alpha( M^{\rm{O}} \circ WH)H^T + \varepsilon } \right)_{ip}}}\\ {H}_{pj} \leftarrow H_{pj} \circ \frac{{\left( {W^TR} \right)_{pj}}}{{\left( {W^T(M^{\it{{\Omega} }} \circ WH) + \alpha W^T(M^{\it{{\rm{O}}}} \circ WH) + \varepsilon } \right)_{pj}}}$$

Following the guidelines to implement NMF^57^, a small number *ε* = 10^−16^ was added to the denominators in Eq. (4) to prevent division by zero, and we initialised *W* and *H* as random dense matrices uniformly distributed in the range [0, 0.1]. Furthermore, to avoid the well-known degeneracy^30^ associated with the invariance *WH* under the transformation $W \to W{\Lambda}$ and $H \to {\Lambda} ^{ - 1}H$, for a diagonal matrix ${\Lambda}$, we normalised *H* at each iteration as follows:

5 $$H_{pj} \leftarrow \frac{{H_{pj}}}{{||{\mathbf{h}}_p||_F}},$$

where **h**_*p*_ denotes the vector corresponding to the *p*th row in *H*.

The stopping criteria of our algorithm was based on the maximum tolerance of the relative change in the elements of *W* and *H*. The default value was TolX < 10^−3^, which occurred typically in about 2,000 iterations for *k* = 10.

We proved that the iterative application of Eq. (4) converges to a local optimal solution point by showing that the multiplicative learning rule satisfies the KKT supplementary conditions of convergence (proof in Supplementary Note 3). The algorithm implemented in Matlab R2018a is provided (see ‘Code availability’).

### Cross-validation procedure and model selection

We randomly selected 10% of the associations in *R*, and these were set aside for testing (held-out test set). We then used a tenfold cross-validation procedure on the remaining 90% of the associations for setting the model parameters *k* and *α*. To do this, we framed the problem as simultaneously predicting the frequency classes and the presence/absence of the associations. Therefore, we used two evaluation metrics:

- RMSE. To quantify the average performance of our model at predicting the frequency class values.
- AUROC. Following Cami et al.^11^, we used AUROC to quantify the performance of our model at predicting the presence or absence of drug side effect associations.

The overall performance of our model in the cross-validation was quantified using the mean RMSE and AUROC over the tenfolds. To select the model parameters, we first chose *α* based on a good binary classification performance (AUROC) while ensuring a good RMSE (see Supplementary Figs. 5 and 6). We found that a good choice of *α* was 0.05. We then chose the value of *k* that minimised the mean RMSE, which occurred for *k* = 10 (Supplementary Fig. 4).

### Thresholding operation by maximum likelihood estimates

For each validation set in the tenfold cross-validation procedure, we collected the frequency classes and their corresponding predicted scores. Then, for each of the five frequency classes, we fitted a normal kernel smoothing function to the predicted scores and obtained a probability density function (pdf) for each of the five classes. The pdfs built for each frequency class defined boundaries for the classification decision by maximum likelihood. The thresholds obtained were: 1.26, 2.43, 3.25, and 3.93. To set a threshold for the zeros, we used the ROC curve obtained when assessing the binary classification performance in the held-out test set. Given a specificity of 0.57, we obtained a sensitivity of 0.97 for a threshold value of 0.426.

To summarise, given a predicted score *x*, a frequency class was chosen using the following thresholds:

$${\mathrm{Predicted}}\;{\mathrm{frequency}}\;{\mathrm{class}}\;(x) = \left\{ {\begin{array}{*{20}{c}} {{\mathrm{zero}}} \hfill& {{\mathrm{if}}\,x \,<\, 0.426}\hfill \\ {{\mathrm{very}}\;{\mathrm{rare}}} \hfill& {{\mathrm{if}}\,0.426 \,\le\, x \,<\, 1.26} \\ {{\mathrm{rare}}}\hfill & {{\mathrm{if}}\,1.26 \,\le\, x \,<\, 2.43}\hfill \\ {{\mathrm{infrequent}}}\hfill & {{\mathrm{if}}\,2.43 \,\le\, x \,<\, 3.25}\hfill \\ {{\mathrm{frequent}}}\hfill & {{\mathrm{if}}\,3.25 \,\le\, x \,<\, 3.93}\hfill \\ {{\mathrm{very}}\,{\mathrm{frequent}}}\hfill & {{\mathrm{if}}\,x \ge 3.93} \hfill\end{array}} \right.$$

This thresholding operation (see Fig. 2c) was applied in all the experiments that required the prediction of the specific frequency classes.

### Accuracy at predicting the specific frequency classes for single drugs

When evaluating the performance of our method on single drugs, we removed only associations for one drug at a time, using the following procedure: (a) we placed the associations that were removed in a test set and then set the corresponding entries in the matrix *R* used for training to zero; (b) we trained our method with all the remaining associations in the training matrix *R*, with parameters *k* = 10 and *α* = 0.05, and (c) we stored the predicted frequency class. Steps (a)–(c) were repeated for each drug.

The mean accuracy of our model at predicting the frequency classes for single drugs was then calculated by the ratio of the number of correct predicted associations and the total number of associations. The accuracy for the exact or neighbouring classes was calculated similarly.

### The similarity between drug and side effect signatures

The similarity between two drug or side effect signatures was calculated using the cosine similarity over the set of latent features. That is, given two drug signatures ${\mathbf{w}}_i \in {\Bbb R}^{1 \times k}$ and ${\mathbf{w}}_j \in {\Bbb R}^{1 \times k}$ (rows vectors in *W*), the drug signature similarity is given by the dot product of the vectors divided by the product of the norm of each vector.

$$S\left( {{\mathbf{w}}_i,{\mathbf{w}}_j} \right) = \frac{{{\mathbf{w}}_i{\mathbf{w}}_j^T}}{{||{\mathbf{w}}_i||||{\mathbf{w}}_j||}},$$

where $||.||$ indicates the vector norm. The similarity for non-negative signatures ranges from 0 to 1.

### Drug signature similarity to capture molecular and clinical drug activity

In our experiments, we trained our model using all the available data and used the best solution of 10,000 independent runs of our algorithm (the solution is provided in Supplementary Data 9 and 10). We formulated three binary classification problems:

*Drug target experiments*. Drug signature similarities were used as scores to predict the binary labels determined by drugs sharing protein targets (1: shared, 0: otherwise; Supplementary Fig. 22). For 435 drugs (for which we could collect protein targets from Drug Bank), 2808 drug pairs were known to share molecular targets, whereas 91,587 were unknown.

*Drug clinical activity experiment*. Drug signature similarities were used as scores to predict the binary labels determined by drugs sharing an ATC category at a given level of the taxonomy (1: shared, 0: otherwise). We tested the performance of the binary classification using the AUROC at each of the ATC levels. Each of the 759 drugs in our data set is annotated in all the levels of the ATC taxonomy. The binary labels used for the assessment on each ATC category were: (anatomical) 38,711 shared vs 248,950 do not share; (therapeutic) 11,960 shared vs 275,701 do not share; (pharmacological) 5,522 shared vs 282,139 do not share and; and (chemical) 1,736 drug pairs shared vs 285,925 do not share.

*Side effect MedDRA category experiment*. Side effect signature similarities were used as scores to predict the binary labels determined by side effects sharing a MedDRA category at a given level of the hierarchy (1: shared, 0: otherwise). We tested the performance of the binary classification using the AUROC. Each of the 994 side effects in our data set is annotated across three description levels of the MedDRA terminology hierarchy. The binary labels used for the assessment on each MedDRA category were (i) level 1 or System Organ Class: 57,076 side effect pairs shared the same category vs 436,445 that do not, (ii) level 2 or High-Level Group Term: 12,097 vs 481,424 and; (iii) level 3 or High-Level Term: 2312 vs 491,209.

### Reproducibility procedure

Following Alexandrov et al.^40,41^, we used these steps to evaluate the reproducibility of the components of the signatures. (Step 1) Train the model 10,000 times using all the available data in the matrix *R* with the parameters *k* = 10 and *α* = 0.05. Each independent run of the algorithm gives a solution $\{ W^r,H^r\} ,r \in \{ 1,2, \ldots ,10000\}$. (Step 2) Select the 100 solutions that gives the smallest values of the cost function at convergence and aggregate them into the *n* × 1000 matrix *W*^rep^ and the 1000 × *m* matrix *H*^rep^. These aggregation matrices now contain the signatures of 100 independent runs of our algorithm. (Step 3) Apply a partition clustering algorithm on the rows of *W*^rep^ and on the columns of *H*^rep^ using cosine distance as the metric. We used the *k*-means++ algorithm by setting the number of clusters to 10. The reproducibility of the signature component was then measured by the tightness and separation of the clusters obtained. We used the cosine similarity-based average silhouette width^58^ of each cluster as a measure of reproducibility of each component in the signature. The silhouette value ranges from −1 to +1. A value close to +1 indicates that a component is very similar to other components in its cluster but very dissimilar to neighbouring clusters.

### Statistical analysis

One-tailed Wilcoxon sum-rank test significance was used in the reported *P* values. To associate a given drug or side effect category to a given component of the signature, we adjusted the *P* values using the Benjamini–Hochberg method^59^ to keep the overall significance level <0.01.

### Reporting summary

Further information on research design is available in the Nature Research Reporting Summary linked to this article.

## Supplementary information

Supplementary Information

Description of Additional Supplementary Files

Supplementary Data 1

Supplementary Data 2

Supplementary Data 3

Supplementary Data 4

Supplementary Data 5

Supplementary Data 6

Supplementary Data 7

Supplementary Data 8

Supplementary Data 9

Supplementary Data 10

Supplementary Data 11

Supplementary Data 12

Supplementary Data 13

Supplementary Data 14

Supplementary Data 15

Supplementary Data 16

Reporting Summary

## Data Availability

Data availability are as follows: frequency classes of drug side effects (Supplementary Data 1), post-marketing side effects (Supplementary Data 2), curated frequencies of Semagacestat side effects from clinical trials I, II and III (Supplementary Data 3), ATC code for each drug (Supplementary Data 4), MedDRA categories for each side effect (Supplementary Data 5 and 6), and drug routes of administration (Supplementary Data 7). The data can also be accessed at https://paccanarolab.org/drug-signatures. Any other relevant data are available from the authors upon reasonable request.

## Code availability

The code of our matrix decomposition model is provided in https://github.com/paccanarolab/Side-effect-Frequencies.
