# Supplementary material for: Predicting the frequencies of drug side effects
Source: Nat Commun. 2020 Sep 11;11:4575. doi: 10.1038/s41467-020-18305-y (PMC7486409; doi:10.1038/s41467-020-18305-y)
Supplement: Supplementary file 1 — Supplementary Information [file 41467_2020_18305_MOESM1_ESM.pdf]

SUPPLEMENTARY INFORMATION FOR

Predicting the Frequencies of Drug Side Effects

Galeano et al., *Nature Communications*, 2020.

*Corresponding author: alberto.paccanaro@rhul.ac.uk

Supplementary Information includes:

Supplementary Notes **1** to **6**

Supplementary Figures **1** to **25**

Supplementary Tables **1** to **3**

Supplementary Data **1** to **16**

Supplementary References

Table of Contents

Supplementary Note 1. Standardisation of the frequencies. 2
Supplementary Note 2. The data-driven regularisation... 5
Supplementary Note 3. Convergence analysis. 8
Supplementary Note 4: Predicting side effect frequencies for a new drug. 9
Supplementary Note 5: Predicting side effect frequencies for an existing drug. 11
Supplementary Note 6. Chemical diversity of our dataset. 12
Supplementary Figures 14
Supplementary Tables 39
Supplementary References 43

Supplementary Note 1. Standardisation of the frequencies.

We started with 1,556 marketed drugs associated with 4,251 distinct side effect terms as listed in the Side effect Resource Database (SIDER) 4.1[1,2]. We found that around 40% of the drug-side effect associations contain frequency information. Side effect terms in the database were annotated with their Medical Dictionary for Regulatory Activities (MedDRA) v20.0 terminology lowest level term (LLT) and preferred term (PT). In the MedDRA terminology, many LLTs may correspond to the same PT. For instance, the MedDRA LLTs Creatinine increased (C0151578), Blood creatinine increased (C0235431), Serum creatinine increased (C0700225) and Plasma creatinine increased (C0858118) corresponds to the same MedDRA PT Blood creatinine increased (C0235431). Therefore, we only used the PT side effect terms to avoid redundancies in side effect terms.

Side effect frequencies were listed in three different formats: (i) exact frequencies, e.g. 1%; (ii) range of frequencies, e.g. 2-5%; and/or (iii) frequency class e.g. "very rare". Placebo frequencies were also provided in a small number of cases, either as exact or range of frequencies. The frequency format with the largest number of elements was the third one: the frequency classes. Therefore, we used frequency classes to standardise all the other frequency formats and to represent the frequencies of the drug side effects. Exact frequencies can be mapped into frequency classes by using the World Health Organisation (WHO) - Council for International Organizations of Medical Sciences (CIOMS) mapping provided in Supplementary Table 1.

To understand the details of our pre-processing procedure for each subset depicted in Supplementary Figure 1. Let set A denote frequency values (exact or range), set B

the frequency classes (the labels) and set C the placebo frequency (exact or range), then for each of the following subsets, we applied the following procedure:

1. Subset A - (A ∪ B). When only the exact or range frequency was available, we compute the median frequency and then mapped it to a frequency class.
2. Subset B - (A ∪ C). When only the frequency class label was available, we kept the labels but normalised the following terms: very rare, rare, infrequent (or uncommon), frequent (or common) and very frequent (or very common).
3. Subset C - (A ∪ B). We have found 215 pairs for which the placebo frequency was found, but the drug frequency was not listed in the database. We manually checked several pairs to confirm that the drug frequency was indeed missing in the database. We discarded these pairs.
4. Subset A ∩ C. We retained pairs for which the median frequency in the intervention cohort was higher than the median frequency in the placebo cohort. For 2,474 pairs, the median frequency in both groups was comparable, and for 403 out of the 2,474 pairs, the placebo frequency was higher. These associations are likely to be caused by the disease or by the so-called nocebo effect[3], i.e. patients that anticipate a side effect on medication is more likely to report it. We discarded these pairs to avoid possible confounders in the associations.
5. Subset A ∩ B. We compute the median frequency value and mapped it to a frequency class. We also kept the frequency class from set B.
6. Subset B ∩ C. We discarded the placebo frequencies for which no intervention frequency was found. We kept the frequency classes from set B.

7. Subset A ∩ B ∩ C. We kept associations for which the median frequency was higher in the intervention cohort than the placebo cohort. We also kept the frequency classes from set B.

After mapping all the frequency formats into frequency classes, we used Supplementary Table 1 to obtain the integers (or frequency values) corresponding to each frequency class. For around 13% of the associations, we found more than one frequency value for a given drug side effect association due to the multiple intersections in the data. 8% of these frequency classes were inconsistent. These might be due to clinical trials from different indications for the same drug. For these cases, we average the frequency values and then round them to the nearest highest integer. Until here, we have extracted 41,546 frequency classes for 860 marketed drugs with around 1,011 unique side effect terms.

Finally, we only kept drugs with known monotherapy Anatomical Therapeutic and Chemical (ATC) category according to the 2018 World Health Organization (WHO) release. We also kept only side effects with known MedDRA category of disorders. In total, our final dataset contains 37,441 frequency values for 759 drugs and 994 side effect terms.

Supplementary Note 2. The data-driven regularisation.

Let us recall from Methods III, that we learn the matrix decomposition that minimises the following objective function:

$$\underset{W,H}{\text{minimise}}\,\mathcal{L}(\mathrm{W},\mathrm{H}) = \frac{1}{2}\parallel M^{\Omega} \circ (R - WH) \parallel_F^2 + \frac{\alpha}{2} \parallel M^{O} \circ (WH) \parallel_F^2 \tag{1}$$

subject to non-negative constraints $W, H \geq 0$.

where $\|.\|_F^2$ is the Frobenius norm, $\circ$ indicates element-wise matrix multiplication, and $M^{\Omega}, M^{O} \in \mathbb{R}^{n\times m}$ are projection functions to separate the learning for the observed and unobserved entries in R. That is, for every entry (i,j) in the matrix,

$$M_{ij}^{\Omega} = \begin{cases} 1, & R_{ij} \in \{1,2,3,4,5\} \\ 0, & \text{otherwise} \end{cases}$$

and,

$$M_{ij}^{O} = \begin{cases} 1, & R_{ij} \in \{0\} \\ 0, & \text{otherwise} \end{cases}$$

We solved the Supplementary Equation (1) by the following multiplicative learning rule:

$$\begin{aligned} W_{ip} &= W_{ip}\frac{(RH^T)_{ip}}{(M^{\Omega} \circ WH + \alpha(M^{O} \circ WH))\, H^T)_{ip}} \\ H_{pj} &= H_{pj}\frac{(\,W^T R)_{pj}}{(\,W^T(M^{\Omega} \circ WH + \alpha(M^{O} \circ WH)))_{pj}} \end{aligned} \tag{2}$$

Our objective function in Supplementary Equation (1) was built to model different levels of uncertainties associated with disjoint subsets of associations in the data. To understand this, consider that due to the data collection methodology in randomised

clinical trials, drug side effects are determined based on whether the incidence in the intervention group is higher than in the placebo group. The side effects determine in this procedure correspond to the true drug side effect associations that are regarded as of high confidence in the first term of Supplementary Equation (1). However, there are not experimentally validated false drug side effect associations. We know that many of the side effects that are not determined in clinical trials might appear after the drug has been marketed [4,5]. Therefore, there is a higher uncertainty onto whether the zeros in R represent false drug side effect associations or true drug side effect associations that are still unknown. We modelled this uncertainty with the penalisation term α in the second term of Supplementary Equation (1). The problem of partially observing a subset of all the associations between pairs of objects is well documented for many problems in experimental biology, pharmacology and medicine[6], as well as in recommender systems and social networks.

The second term in Supplementary Equation (1) has a regularisation effect on the learned signatures. By changing α between 0 and 1, we can control the sparsity of the learned signatures thus reducing the model complexity. This regularisation effect is unlike any other regularisation terms that have been previously introduced in the literature of machine learning. Typically, in matrix decomposition, regularisation terms are applied to the matrices W and H[7]. For instance, by using L1 or L2 norms. Newer methods also use the nuclear-norm regularization[8,9]. In our model, the intrinsic sparsity in the input matrix R is used to regularise the solution. We refer to this effect as data-driven regularisation.

Our method is also deeply connected to previous formulations of non-negative matrix factorisation, including the NMF algorithm from the seminal work of Lee and Seung[10].

Let us consider the extreme values of α in Supplementary Equation (1). In the case of $\alpha = 1$, our cost function can be more concisely written as:

$$\min_{W,H} \sigma(W,H) = \frac{1}{2} \parallel R - WH \parallel_F^2 \quad (3)$$

$$\text{subject to } W, H \geq 0$$

which is the original NMF formulation[10,11]. In this case, our model achieves the higher sparsity for the signatures. On the other hand, when $\alpha = 0$, our objective function is equivalent to the weighted NMF used in movie recommendation systems[8]. In this case, the learned signatures are dense as only non-zero elements in R are used to fit the model.

Consequently, the distribution of predicted scores, $\hat{R} = WH,$ also changes with α. When α increases from 0 to 1, the distribution of $\hat{R}$ changes from a distribution that resembles the distribution of observed entries in R (e.g. a Gaussian distribution with mean μ and s.t.d. σ) to a zero-inflated distribution (with a large number of scores close to zero). This characteristic of our model provides great flexibility because it allows for a balance between *observation* and *expectation*. For instance, If the expectation is that most of the unobserved associations in R should remain zero values, then α should be set to a value close to 1.

Supplementary Note 3. Convergence analysis.

Supplementary Theorem 1. *The functional $\mathcal{L}(W, H)$ in Supplementary Equation (1) converges to a local minimum under the update rules in Supplementary Equation (2).*

Proof. From the theory of constrained optimization[12], we know that to prove Supplementary Theorem 1, we need to show that at convergence, the solution satisfies the well-known Karush-Khun-Tucker (KKT) complementary conditions:

$$\left(\frac{\partial \mathcal{L}}{\partial \mathrm{W}}\right)_{ip} W_{ip} \;=\; 0, \left(\frac{\partial \mathcal{L}}{\partial \mathrm{H}}\right)_{pj} H_{pj} \;=\; 0 \qquad (4)$$

where $i \in \{1, \ldots, n\}$, indexes the n drugs, $j \in \{1, \ldots, m\}$ indexes the m side effects and $p \in \{1, \ldots, k\}$, indexes the k latent features.

The gradients of Equation (1) can be written in matrix form, as follow:

$$\frac{\partial \mathcal{L}}{\partial W} = -\left(R - M^{\Omega} \circ WH\right) H^T + \alpha(M^O \circ WH)H^T$$
$$\frac{\partial \mathcal{L}}{\partial H} = -W^T(R - M^{\Omega} \circ WH) + \alpha W^T \alpha(M^O \circ WH) \qquad (5)$$

At a local minimum, $\mathrm{W} = W^*$ and $\mathrm{H} = H^*$ must satisfy the KKT conditions in Supplementary Equation (4). Replacing the gradients (5) in (4):

$$\left(RH^T\right)_{ip} W_{ip} - \left(\left(M^{\Omega} \circ WH + \alpha(M^O \circ WH)\right)H^T\right)_{ip} W_{ip} \;=\; 0$$
$$\left(W^T R\right)_{pj} H_{pj} - \left(W^T(M^{\Omega} \circ WH + \alpha(M^O \circ WH))\right)_{pj} H_{pj} \;=\; 0 \qquad (6)$$

By re-ordering equations in (6), we obtained:

$$W_{ip} = W_{ip} \frac{(RH^T)_{ip}}{(M^{\Omega} \circ WH + \alpha(M^O \circ WH))\, H^T)_{ip}}$$

$$H_{pj} = H_{pj} \frac{(W^T R)_{pj}}{(W^T(M^{\Omega} \circ WH + \alpha(M^O \circ WH)))_{pj}}$$

which are identical to our multiplicative learning rules in Supplementary Equation (2). Therefore, the algorithm satisfies KKT conditions and converges to a local minimum.

■

Supplementary Note 4: Predicting side effect frequencies for a new drug.

Our method can be applied for a new drug that is not currently in our dataset, as long as few side effect frequencies are available. Our algorithm needs a few side effect frequencies to start with. For instance, after gathering initial results from a small-size phase I clinical trials, as showcased by our case-study of Semagacestat.

The step-by-step procedure to predict frequencies of side effects for a drug that is not currently in our dataset is as follows (see Supplementary Figure 14):

- Step 1: calculate the exact frequencies of side effects in intervention and placebo groups.
- Step 2: keep only those associations in which the frequency in the intervention group is higher than in the placebo group.
- Step 3: use the mapping provided in Supplementary Table 1 to obtain the side effect frequency classes (the numbers from 1 to 5).
- Step 4: add a new row into our data matrix R for the new drug and then incorporate the values that encode each side effect frequency class. By adding the new drug at the end of the rows of R, we can obtain a new matrix of $(n+1) \times m$ $R' = [R,\ r_{new}]$, where r_{new} correspond to an $1 \times m$ row vector containing the frequency classes of the new drug.

- *Step 5*: run our multiplicative learning algorithm using R' as input. Use the default parameters: number of features $k = 10$, and confidence on the zeros $\alpha = 0.05$. Our algorithm will return two new matrices: an $(n + 1) \times k$ drug signature matrix W', and a $k \times m$ side effect signature matrix H'. The algorithm could be also run multiple independent times to obtain a solution that gives the smallest value of the objective function at convergence.
- *Step 6*: Predict the frequency classes by thresholding the scores in the last row of R' (this is the row that corresponds to the new drug). Thresholds are provided in Methods V.
- *Step 7 (optional)*: To investigate the potential off-target activity across anatomical systems using the signatures, it is required to analyse the newly learned signatures. Notice that since our studied solution summarised in Table 1 in the main manuscript corresponds to a different initial starting point, the components of the newly learned signatures will not match the biological interpretation of the studied solution in our manuscript. To match a specific component of the new signature to our studied solution, we recommend using our procedure in Methods IX. The idea is to cluster together, the components of the new signatures (obtained from multiple independent runs of the algorithm, e.g. 1,00 best solutions out of 10,000), and our studied solution as a reference (we provide the studied solution in Supplementary Data 9-10). The components of the new signatures can be then reliably matched to the components of the studied signatures by looking at which cluster the components belongs to. There are other algorithms in the literature of graph theory, that might be useful for this task when formulated as a graph matching problem between two sets of nodes[13].

Supplementary Note 5: Predicting side effect frequencies for an existing drug.

Our method can also be applied to predict the frequencies of drug side effects for existing drugs. We visualised the three following scenarios: (A) the drug is already in our dataset; (B) the drug is already in the dataset but more data on the frequencies of side effects is available for integration (e.g. from other proprietary sources); or (C) the drug has known side effect frequencies but it is not currently in our dataset. We explain the procedure for predicting the frequencies of side effects for a drug of interest in each case below:

- **Case A**: all the predictions for the drugs in our dataset are provided in Supplementary Data 8.
- **Case B**: The step-by-step procedure is as follows:
    - *Step 1*: use Supplementary Table 1 to obtain the frequency classes for the new associations.
    - *Step 2*: add the new associations in the corresponding row of R.
    - *Step 3*: run our multiplicative learning algorithm using the updated R as input. Use the default parameters: number of features $k = 10$, and confidence on the zeros $\alpha = 0.05$. Our algorithm will return two new matrices: an $n \times k$ drug signature matrix W, and a $k \times m$ side effect signature matrix H. The algorithm could be also run multiple independent times to obtain a solution that gives the smallest value of the objective function at convergence.

- *Step 4*: Predict the frequency classes by thresholding the scores in the row of R that correspond to the drug of interest. Thresholds are provided in Methods V.
- *Step 5 (optional)*: To investigate the potential off-target activity across anatomical systems an analysis of the new drug's signature and side effect signatures is required. Notice that since the studied solution in the main manuscript (see Table 1) corresponds to a different initial starting point, the components of the signatures will not match exactly the biological interpretation that we provided in the manuscript. To do this matching, we recommend following our procedure in Methods IX by including in the clustering procedure the interpreted solution provided in Supplementary Data 9-10. The components of the new signatures can be then reliably matched to the components of the interpreted signatures and thus the biological meaning exploited to formulate hypotheses.

- **Case C**: follow the procedure described in Supplementary Note 4.

Supplementary Note 6. Chemical diversity of our dataset.

When removing chemically redundant drugs from our dataset, we would like to know whether the threshold chosen for the chemical similarity is low or high. Yet, the chemical similarity value often depends on the specific chemical fingerprint chosen. Therefore, to assess if our set of drugs is enriched in chemically similar or dissimilar drugs, we need to compare the distribution of chemical similarities in our set of drugs to a dataset that we know is chemically diverse. To make this task feasible and relevant to our application, we focused on finding a reference set that it is

representative of the chemotypes that have reached clinical development, including approved drugs and compounds in distinct phases of clinical trials.

We found a recently published dataset that met our criteria: the Drug Repositioning Hub database from the Broad Institute[14]. The Drug Repositioning Hub (RepoHub, hereafter) has 6,806 compounds with known SMILES notations. Importantly, in their original paper, Corsello et al. [14] assessed the structural diversity of the RepoHub compounds by modelling and representing the chemical space as a self-organising map with 16×16 hexagonal cells. Of the 256 clusters obtained by self-organising maps, around 250 had at least one compound in it, reflecting the chemical diversity of the RepoHub library (the Supplementary Figure 1a in Corsello et al. [14] illustrates very nicely the structural diversity of this set of compounds).

To compare our set of drugs to this reference set, we employed chemical fingerprints from the SMILES notations using the RDKit Fingerprint (Methods I in the main manuscript). We calculated the pairwise similarities among 754 drugs from our dataset (a total of 283,881 pairwise similarities) and among the 6,806 RepoHub compounds (a total of 23,157,415 pairwise similarities).

Supplementary Figure 18 below compares the normalised histograms of the distributions of chemical similarity scores for the RepoHub reference set of compounds (mean and s.t.d. chemical similarity, 0.226±0.118) and for our set of drugs (mean and s.t.d. chemical similarity, 0.249±0.127). Although there are 80 times more pairwise similarities in the RepoHub reference set than in our set of drugs, there is only a 2.3% difference in the mean Tanimoto chemical similarity between them. A Tanimoto chemical similarity score of 0.6 is in the 99.9th percentile of the reference distribution. Therefore, drugs whose chemical similarity is below 0.6 can be regarded as chemically dissimilar.

Supplementary Figures

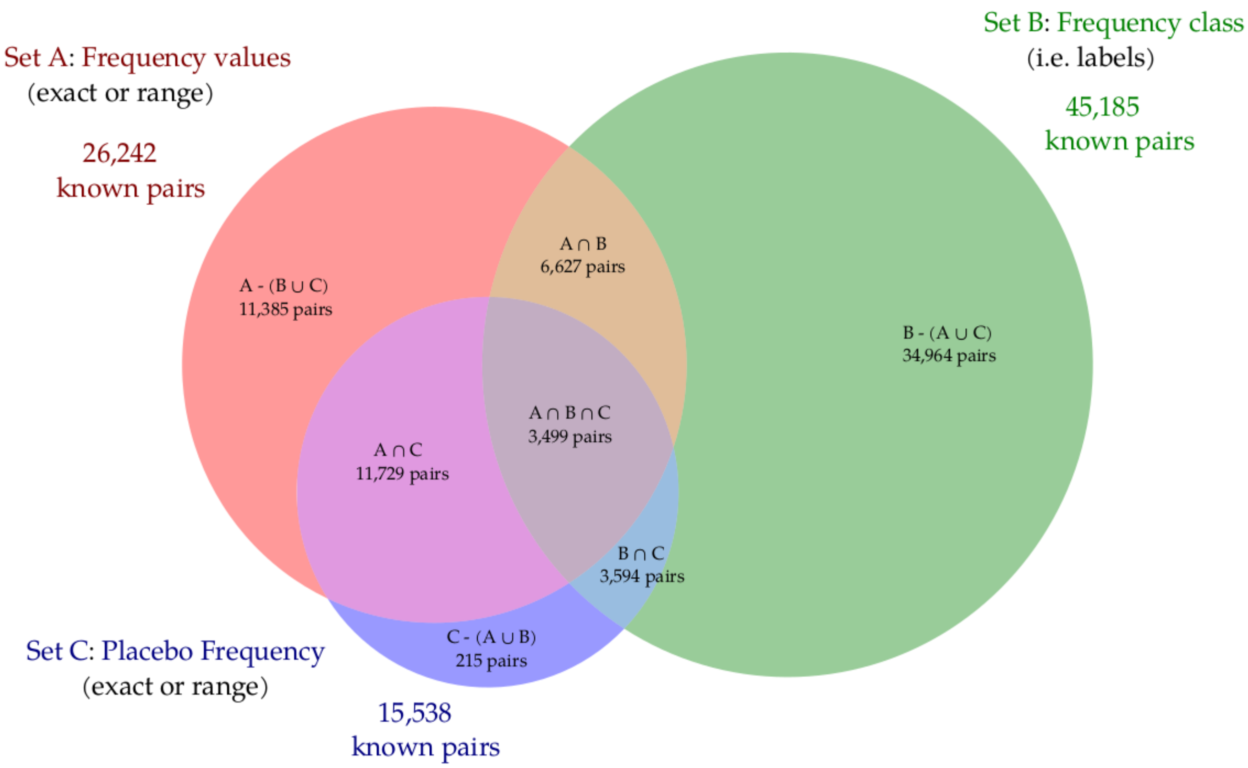

Supplementary Figure 1. Venn diagram depicting the different formats for the drug side effect frequencies in SIDER 4.1. In total, 68,514 pairs were found with frequency information. There are three overlapping sets of data formats. Set A: contains drug exact (e.g. 1%) and range frequency (e.g. 2-5%); set B contains frequency classes (e.g. very rare), and set C contains the exact and range placebo frequencies. The size of the circles is proportional to the number of drug-side effect pairs in each set.

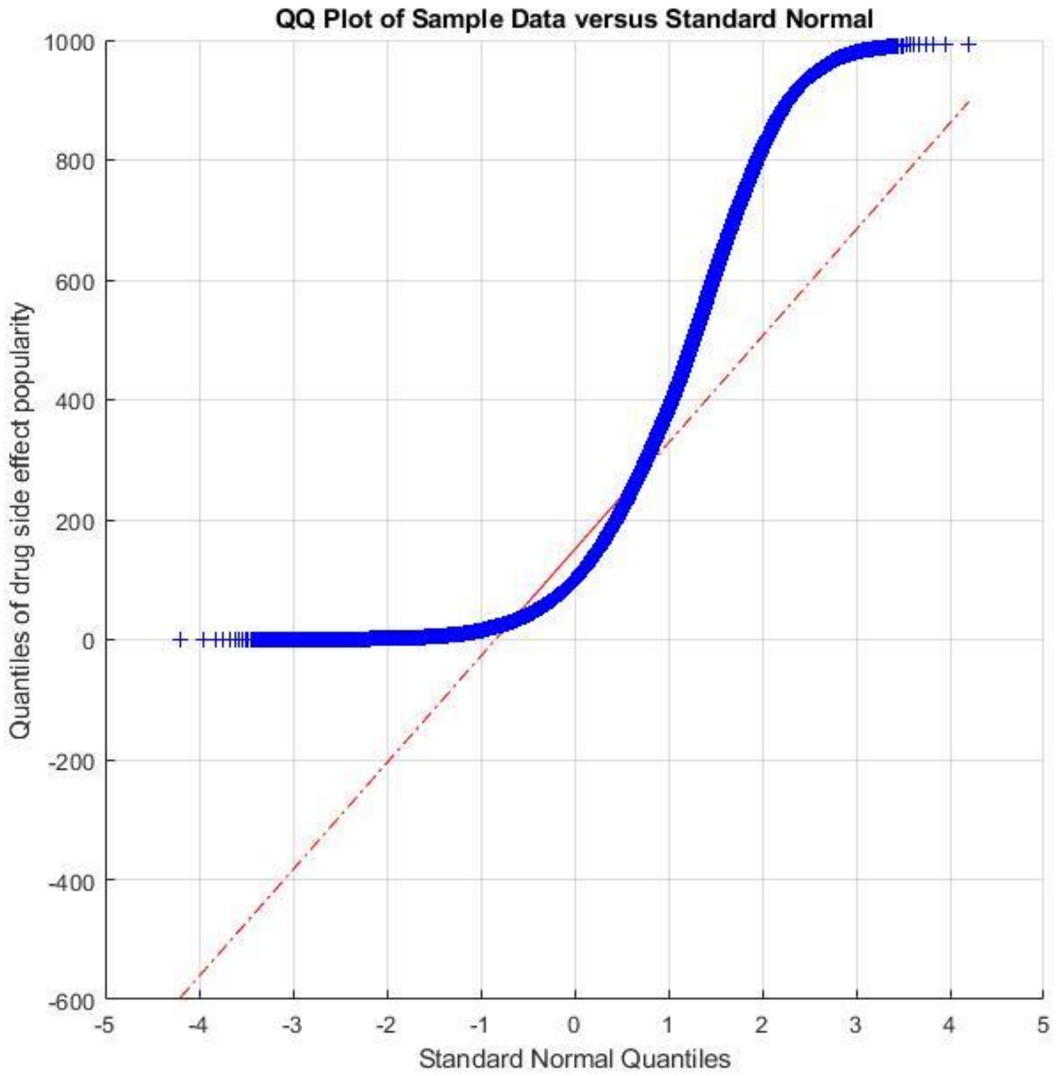

Supplementary Figure 2. QQ plot of side effects distribution. The plot shows the quantile-quantile distribution of the data versus the theoretical quantile value from a normal distribution. The kurtosis is a measure of how outlier-prone the distribution is. The kurtosis of the side effects distribution is 5.04; in comparison, the kurtosis of a normal distribution is 3.

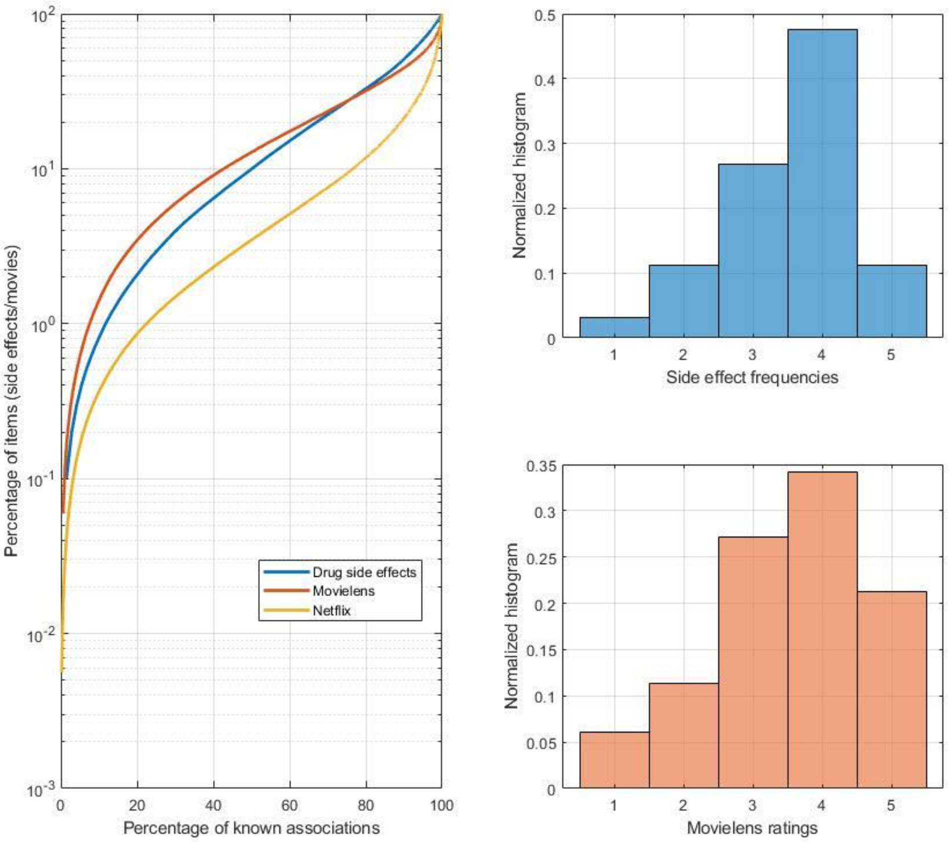

Supplementary Figure 3. The heavy-tailed distribution of drug side effects and two movie datasets. *(*Left*)* Side effects and movies (items) are ordered according to popularity, most popular at the bottom. Side effects and movie datasets tend to have a few popular items containing more than 20% of the associations (usually known as short head). However, most items reside in the long tail of the distribution, populated with items (side effects or movies) with fewer associations. The Movielens dataset contains 943 users and 1682 movies with 100K associations (~6.3% density). The Netflix dataset contains 480,189 users and 17,770 movies with 100M associations (~1.17% density). The density of the Movielens dataset is more comparable to our dataset of drug side effects (~4.96% density). *(*Right*)* Distribution of rating values for drug side effect frequency and the rating values in the Movielens dataset. The distribution of frequency values comes from a normal distribution (Chi-square goodness-of-fit Significance, p-value < 2.23e-308) and it is very similar to the distribution of ratings in Movielens (Kolmogorov-Smirnov Significance, 2.51e-233).

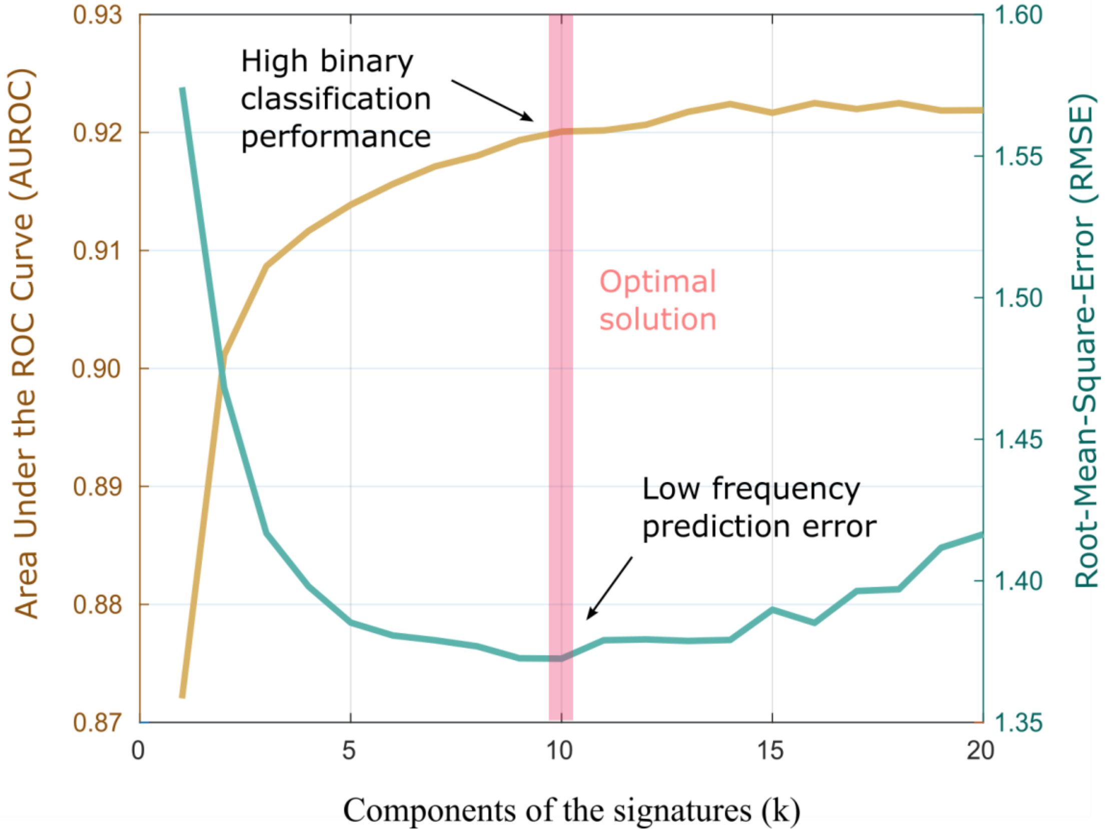

Supplementary Figure 4. Model selection. Selection of the optimal number of latent features (or signatures components) based on the RMSE-AUROC trade-off. Here $\alpha = 0.05$.

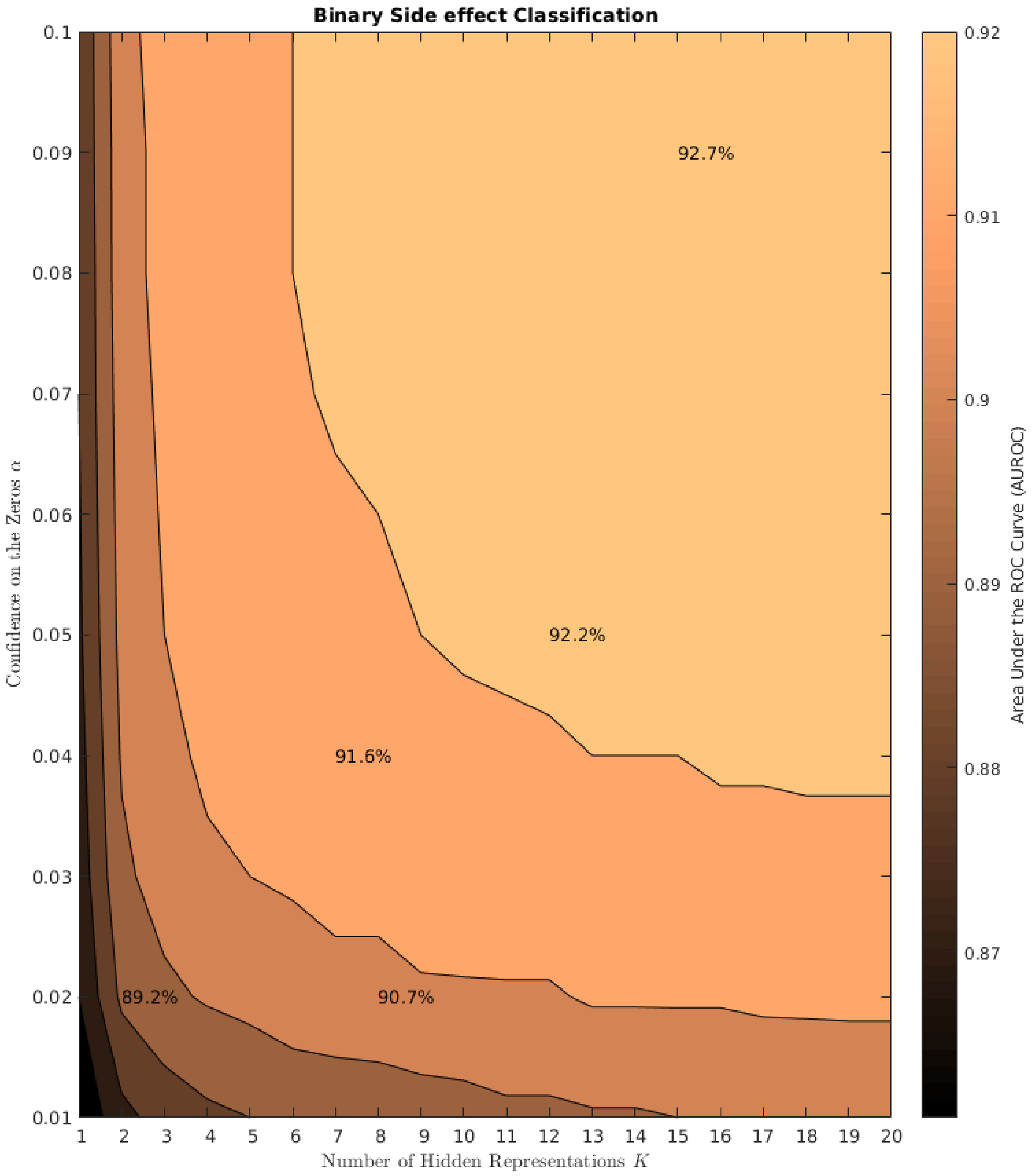

Supplementary Figure 5. Contour plot of mean AUROC of the ten-fold cross-validation performance for the binary side effect classification problem. The higher the AUROC, the better we can correctly identify true associations. The performance is divided, for clarity, into nine contour levels for varying values of the number of latent features (k) and the confidence in the zeros (α).

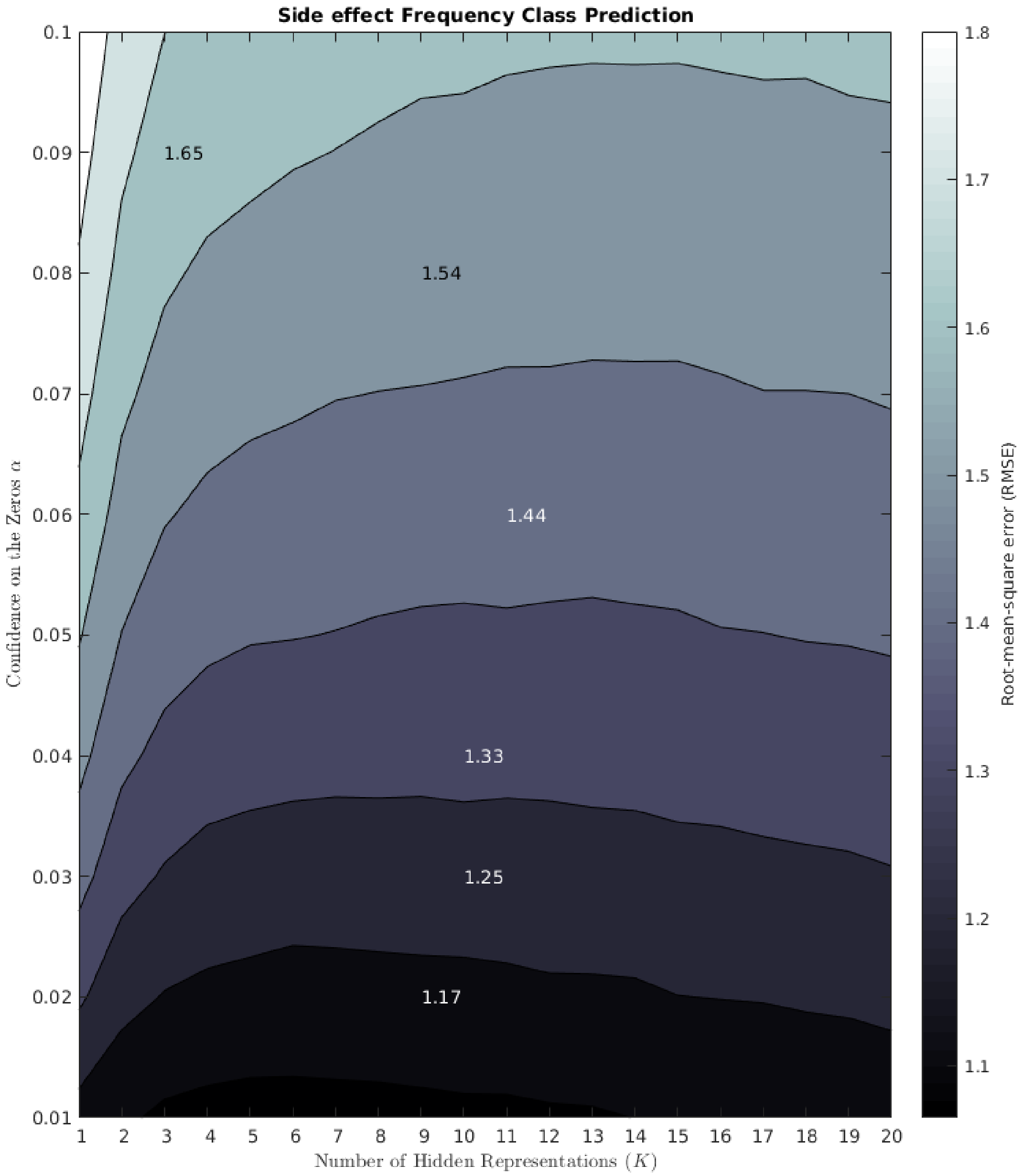

Supplementary Figure 6. Contour plot of mean RMSE of the ten-fold cross-validation performance for the side effect frequency class value prediction problem. The smaller the RMSE, the better we can predict the true frequency value of the drug side effects. The performance is divided for clarity into nine contour levels for varying values of the number of latent features (k) and the confidence in the zeros (α).

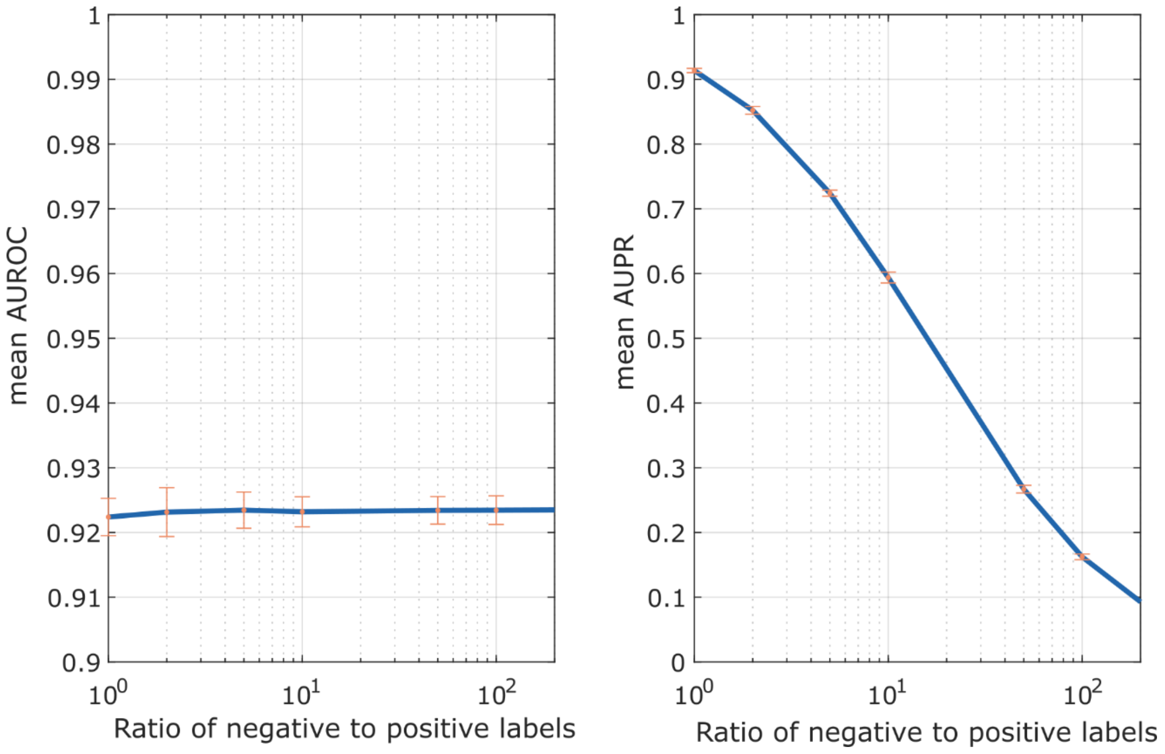

Supplementary Figure 7. Mean and standard deviation for AUROC and AUPR metrics computed using ten-fold cross-validation for varying values of negative to positive label ratios. Here, positive and negative classes indicates the presence or absence of drug side effects, respectively.

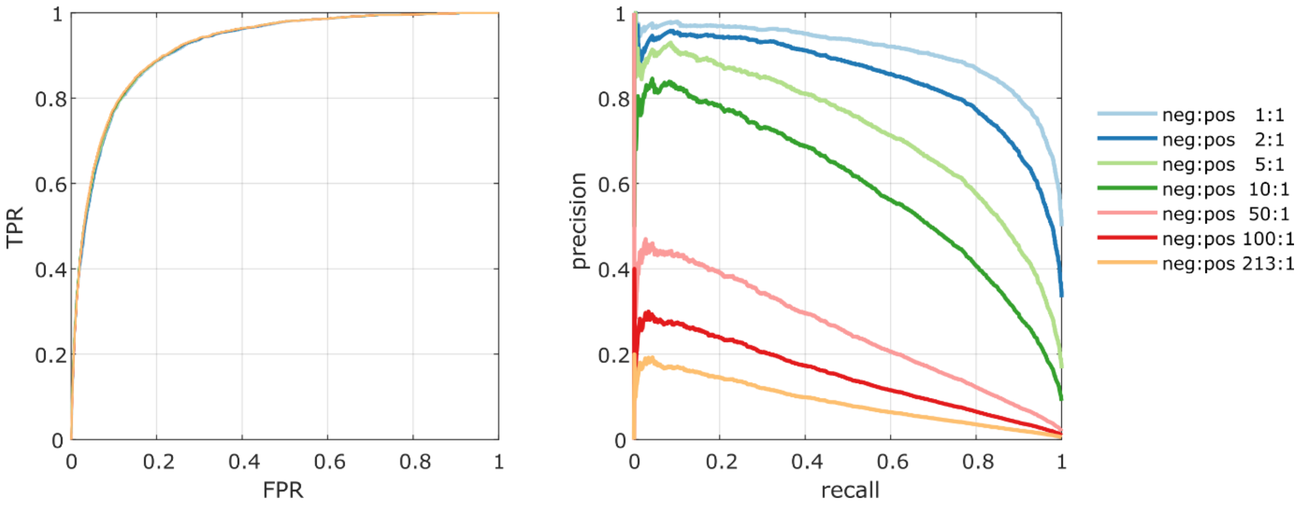

Supplementary Figure 8. Binary classification performance in the held-out test set for varying ratio of negative to positive labels. (Left) ROC curves. All the curves overlap. (Right) PR. A negative to positive ratio (neg:pos in the figure) of 1:1 indicates balance number of positives and negative classes in the test set. Here, positive and negative classes indicates the “presence” or absence of drug side effects, respectively.

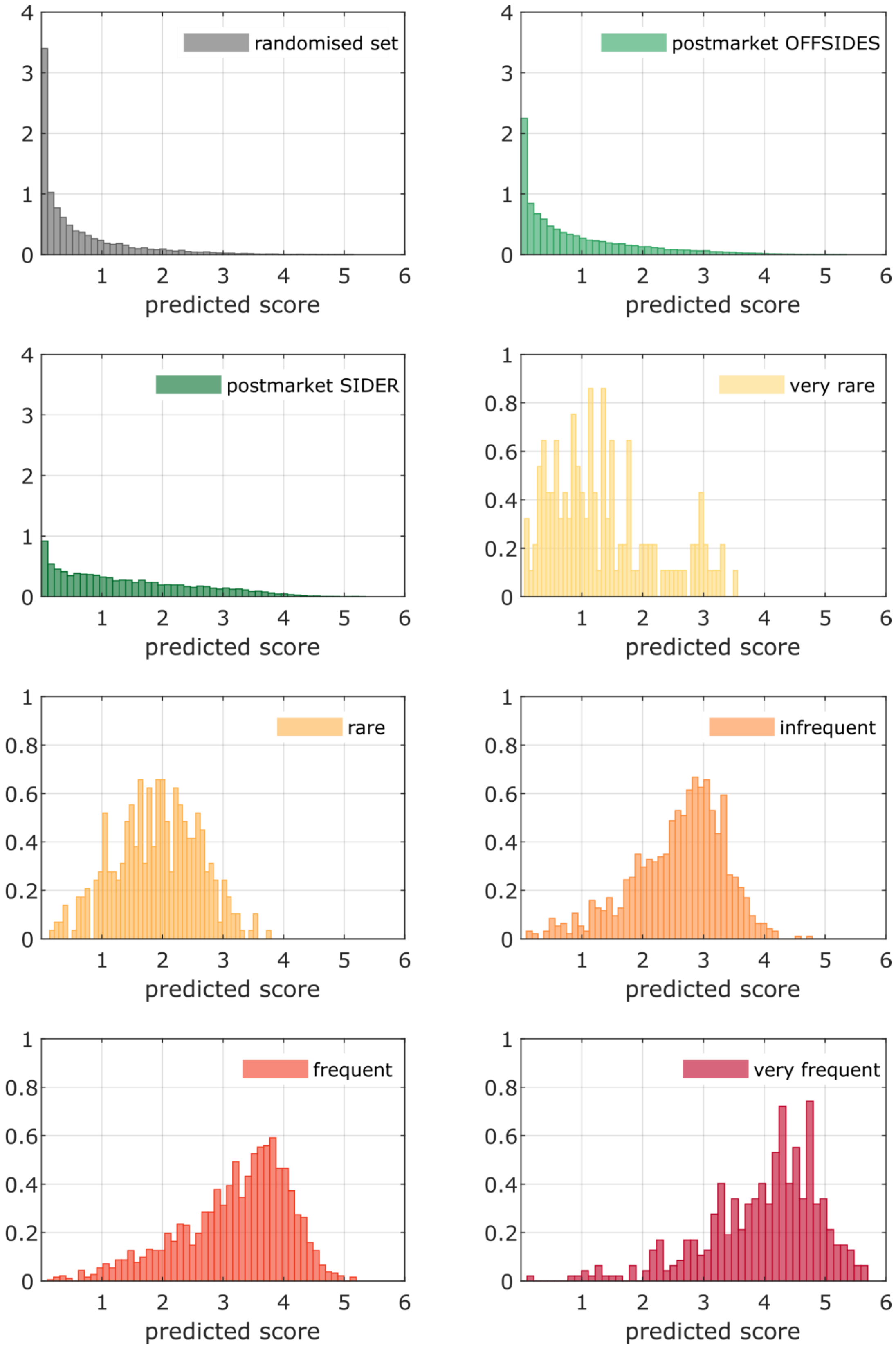

Supplementary Figure 9. Normalised histograms of the distribution of predicted scores obtained for the randomised, held-out and post-marketing test sets.

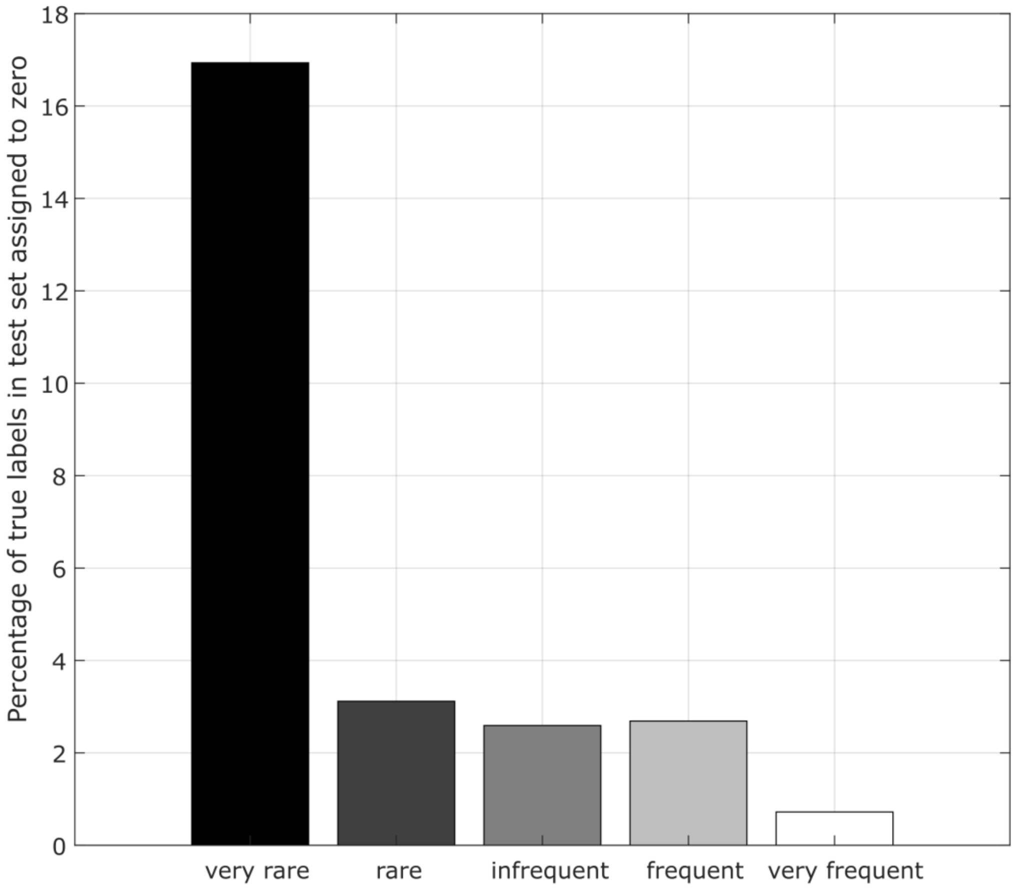

Supplementary Figure 10. Percentage of true labels in the held-out test set assigned to zero for each of the side effect frequency classes. Around 15% of the very rare drug side effects were assigned to zeros, likely driven by the few numbers of labels in the dataset. However, less than 3% of the associations belonging to the other classes were incorrectly classified as false drug side effect.

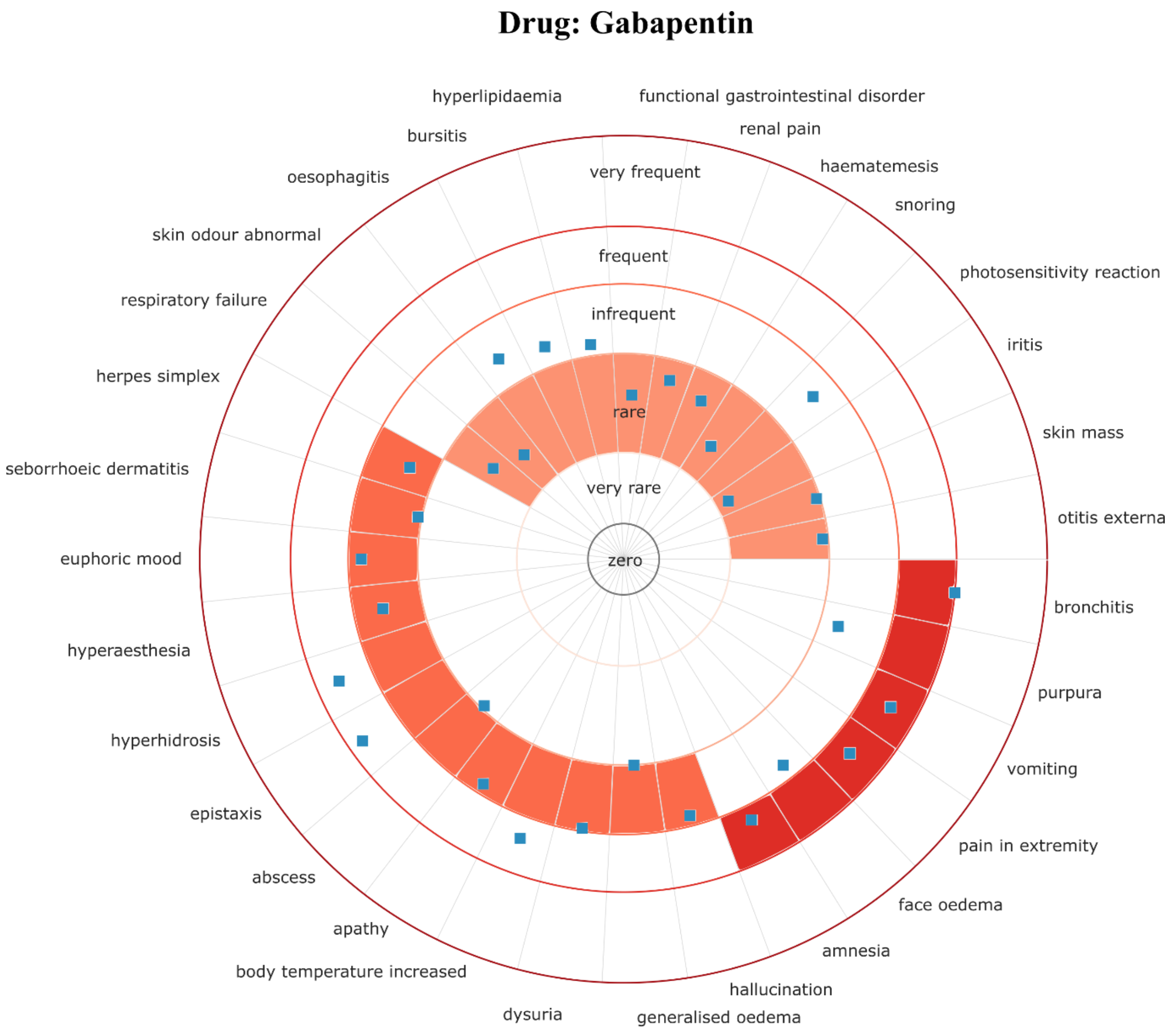

Supplementary Figure 11. Predictions for all the side effects produced by the drug Gabapentin from the held-out test set. The thirty-one predictions in the test set for the anticonvulsant drug Gabapentin are shown around polar plots, each in a dedicated sector. Concentric circles between frequency classes correspond to thresholds learned by maximum likelihood. The correct class for each association is coloured in each circular sector, while predicted scores are shown as blue squares.

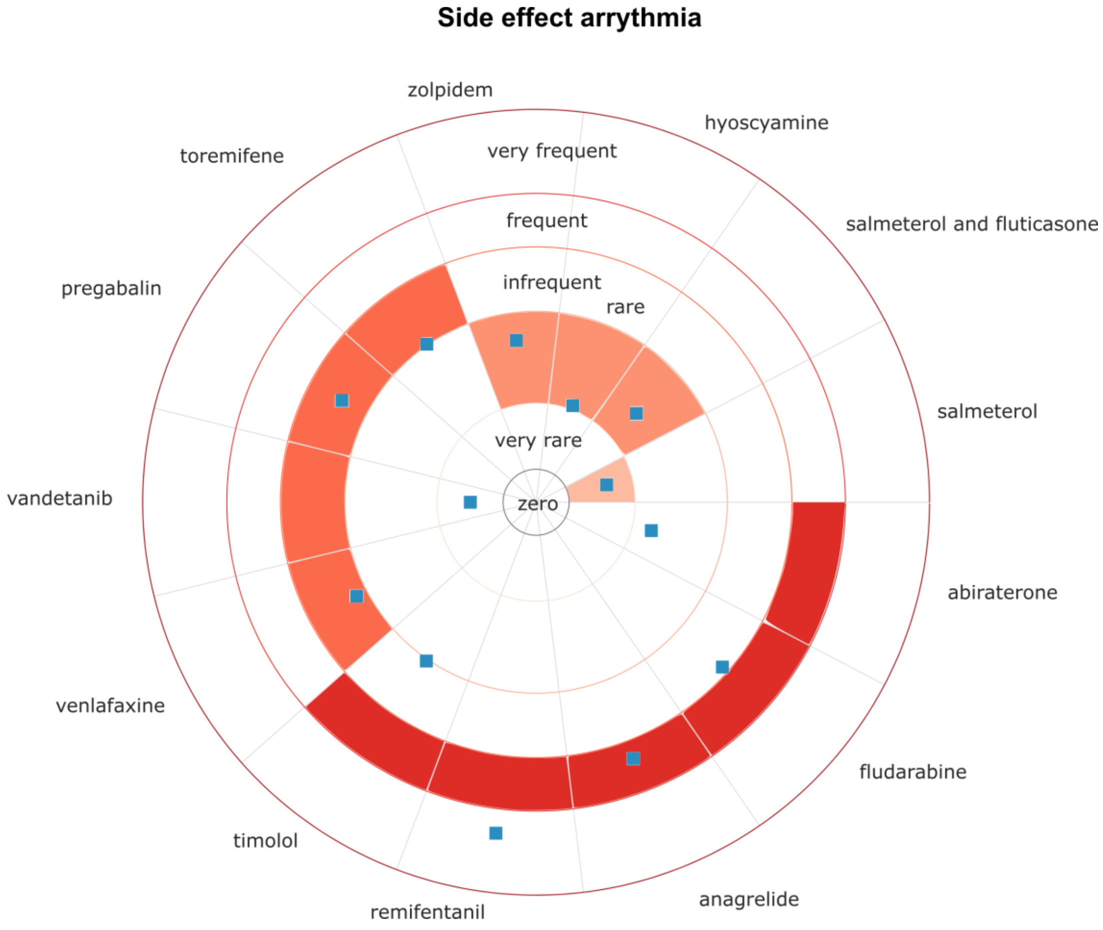

Supplementary Figure 12. Predictions for all the drugs that produce arrhythmia from the held-out test set. The thirteen predictions in the test set for the cardiovascular side effect arrhythmia are shown around polar plots, each in a dedicated sector. Concentric circles between frequency classes correspond to thresholds learned by maximum likelihood. The correct class for each association is coloured in each circular sector while predicted scores are shown as blue squares.

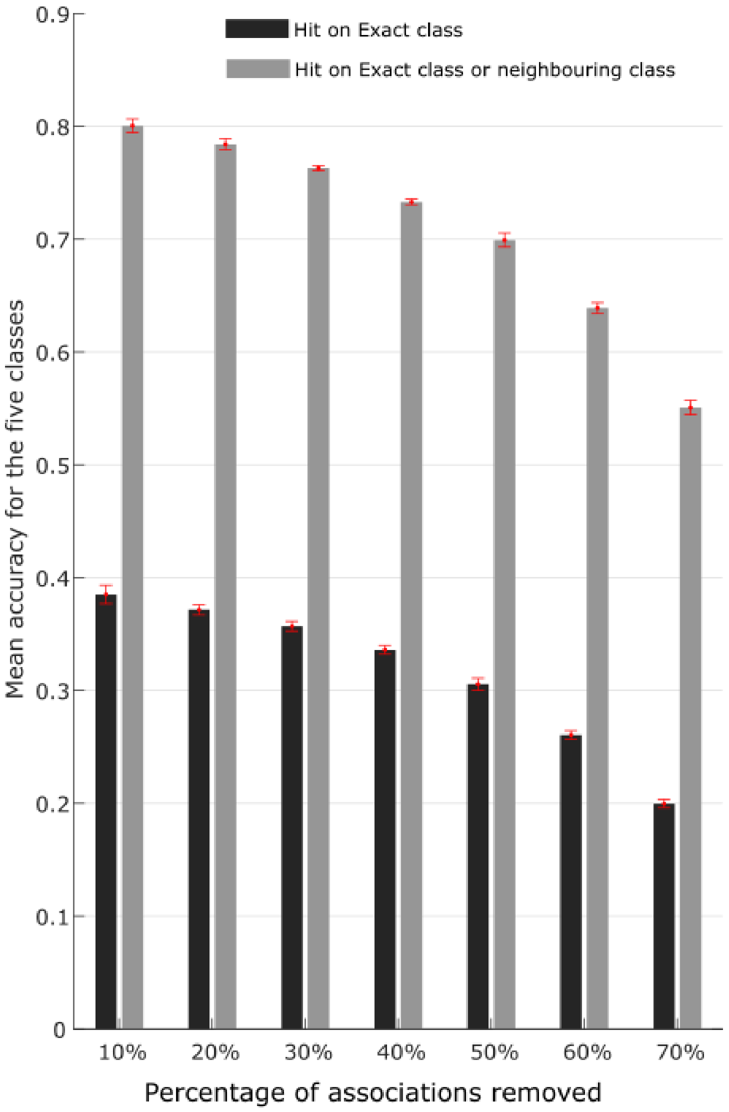

Supplementary Figure 13. Mean accuracy of our method for the five frequency classes when removing varying percentages of associations from the matrix R. (Black bars) Mean accuracy on the exact class; (Grey bars) Mean accuracy on the exact or neighbouring class. Errors bars in red indicate the standard deviation over ten independent repetitions.

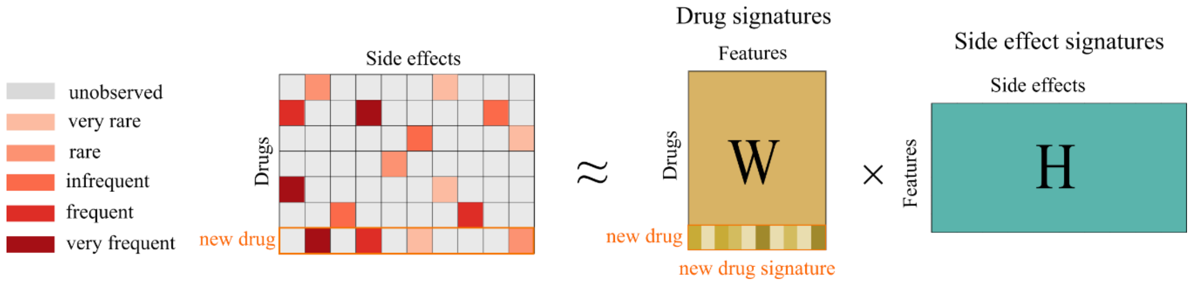

Supplementary Figure 14. Learning the drug signature for a new drug. The procedure to generate predictions and thus signatures for a new drug requires to refit our model to an expanded data matrix. First, the new drug information is incorporated into a new row of data matrix R. By adding the new drug at the end of the rows of R, we can obtain a new matrix of $(n+1) \times m$ $R' = [R,\ r_{new}]$, where r_{new} correspond to an $1 \times m$ row vector containing the frequency classes of the new drug. Second, our multiplicative learning algorithm needs to be applied to learn the new model $\widehat{R}' = WH$, that models R'. The row in W corresponding to the new drug contains the signature vector of the new drug.

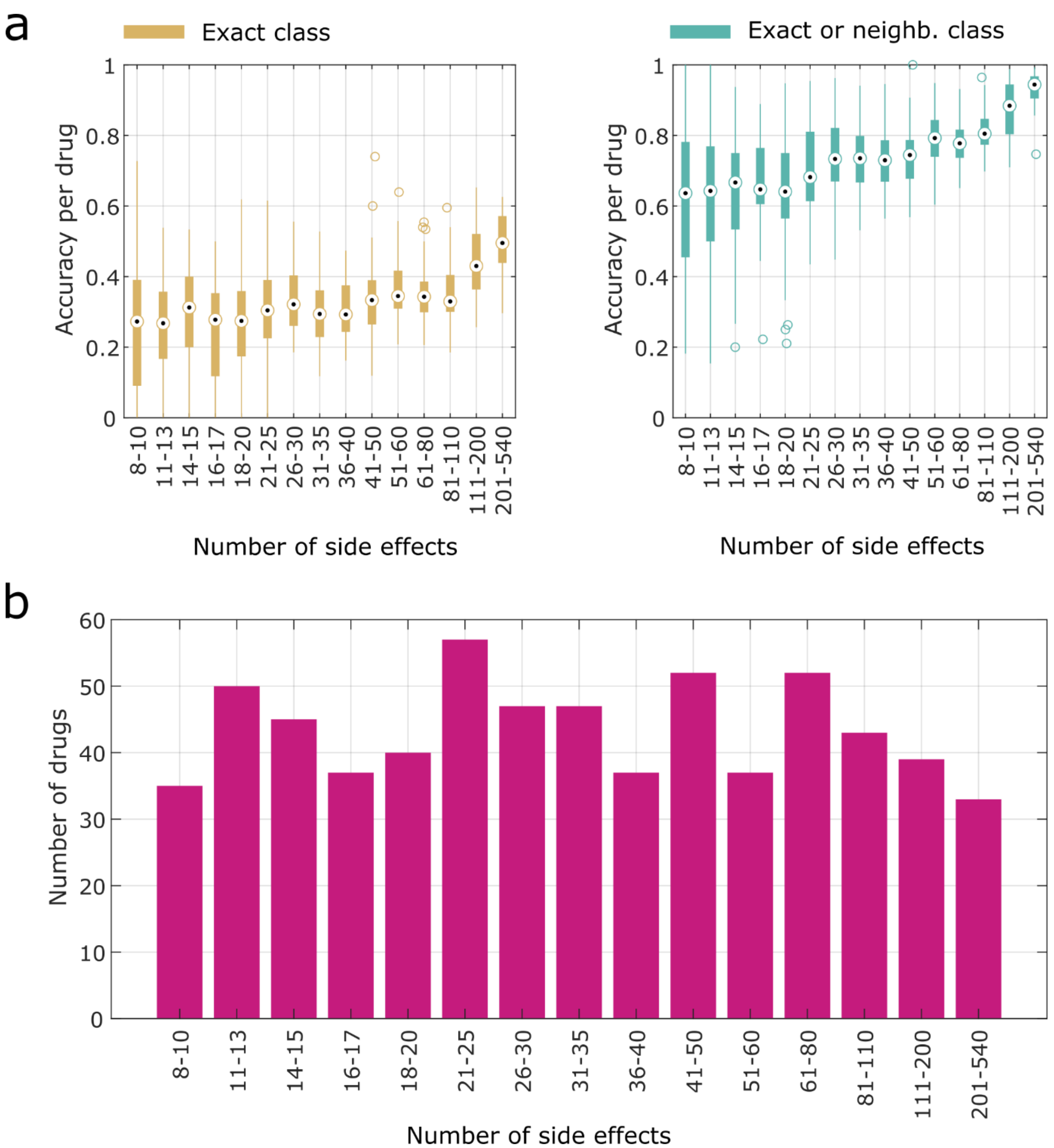

Supplementary Figure 15. Accuracy vs total number of known side effects per drug. (a-Left) Accuracy for the exact class; (a-Right) Accuracy for the exact class or neighbouring class. (b) Number of drugs for each bin of the number of side effects.

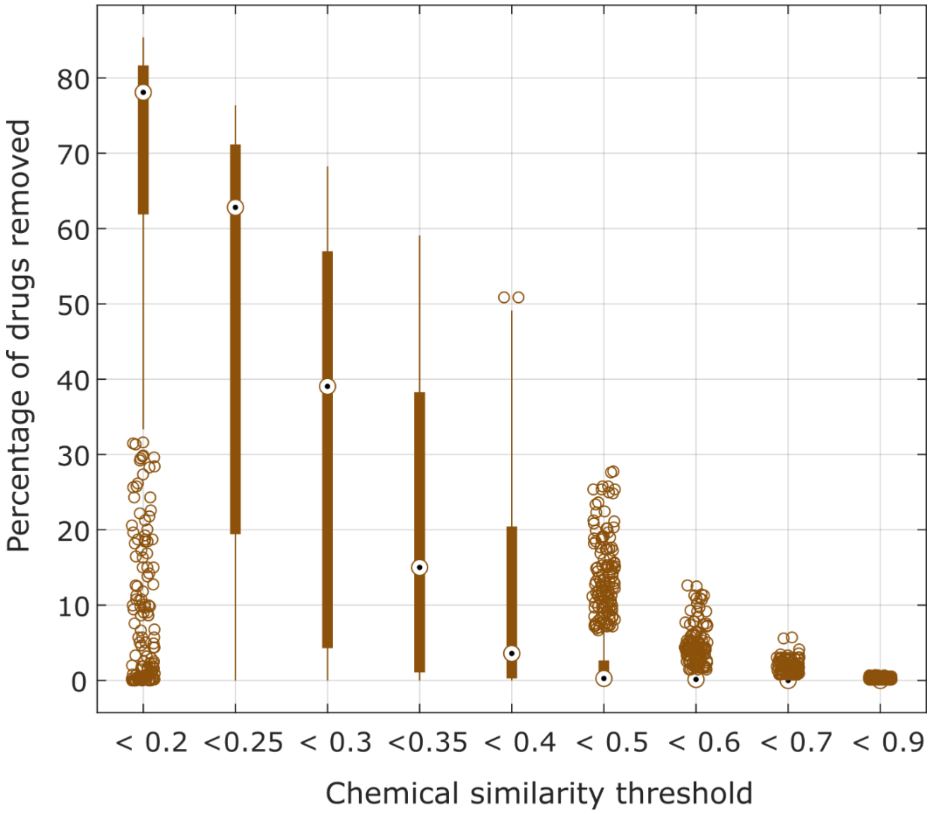

Supplementary Figure 16. Percentage of drugs that are removed from training set for a given threshold of the chemical similarity.

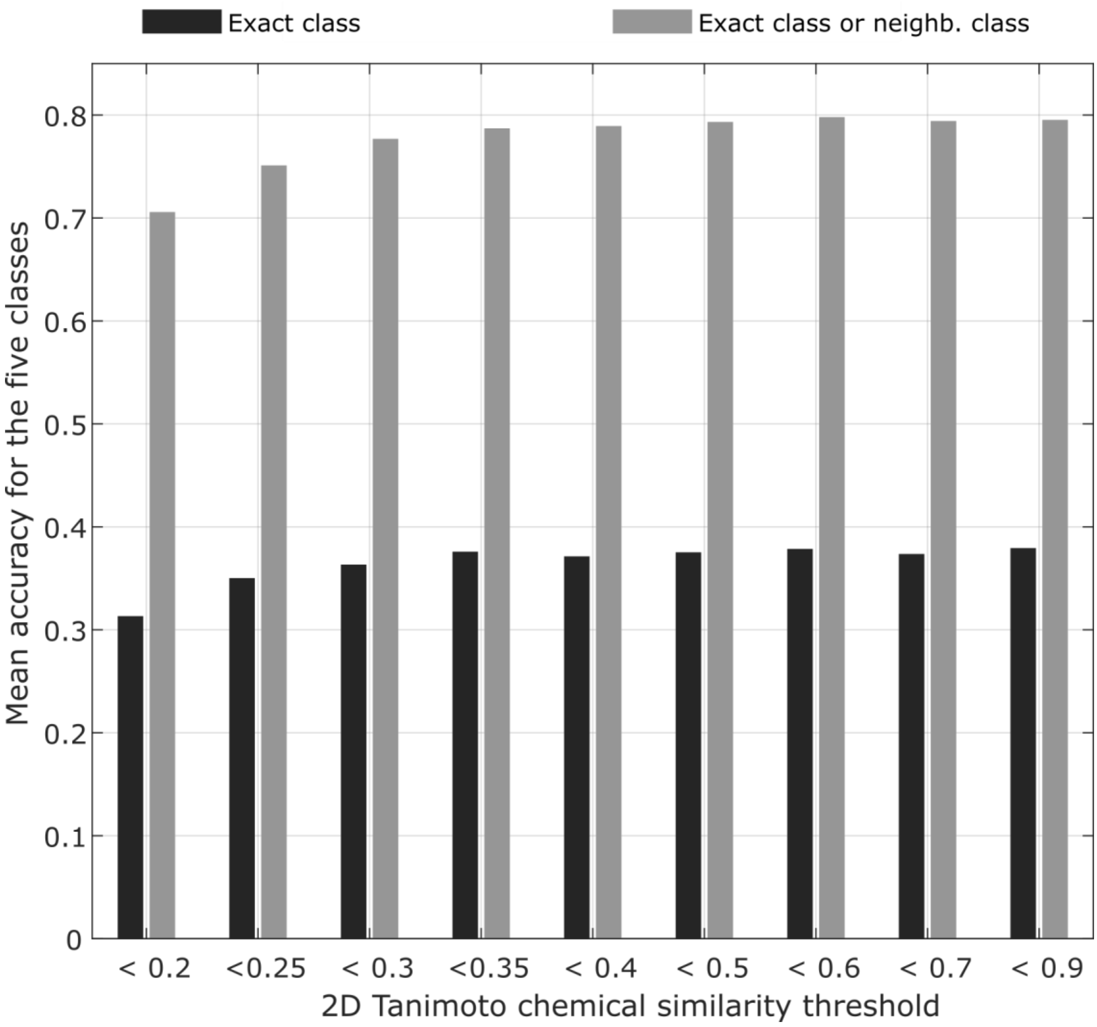

Supplementary Figure 17. Mean accuracy at predicting the frequencies of drug side effects when removing chemically similar drugs. For each of the 754 drugs, 10% of randomly chosen associations were removed for testing. Only drugs below a given 2D chemical similarity threshold were kept for training. Ten independent repetitions were performed.

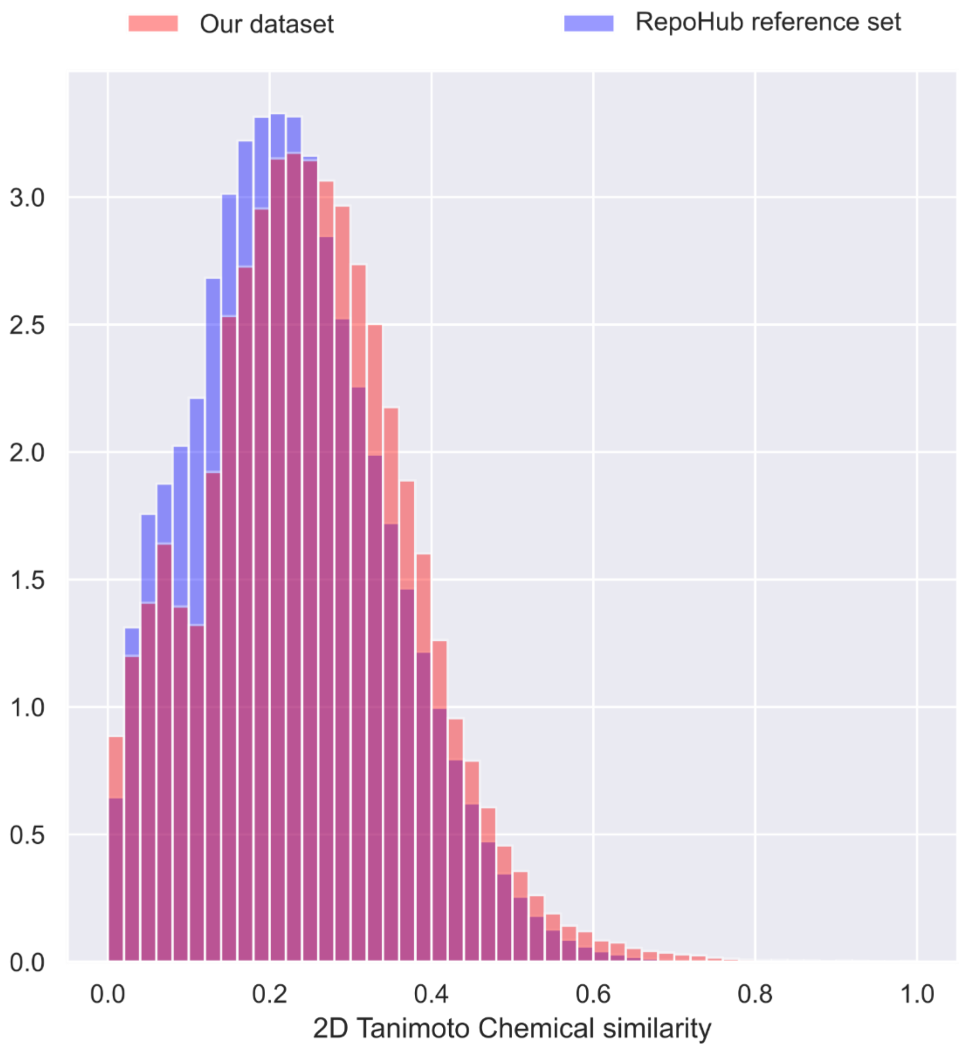

Supplementary Figure 18. Normalised histogram of the distributions of 2D Tanimoto chemical similarities between (*Blue*) 6,806 compounds from the Drug Repositioning Hub Library; (*Red*) 754 drugs used in our study.

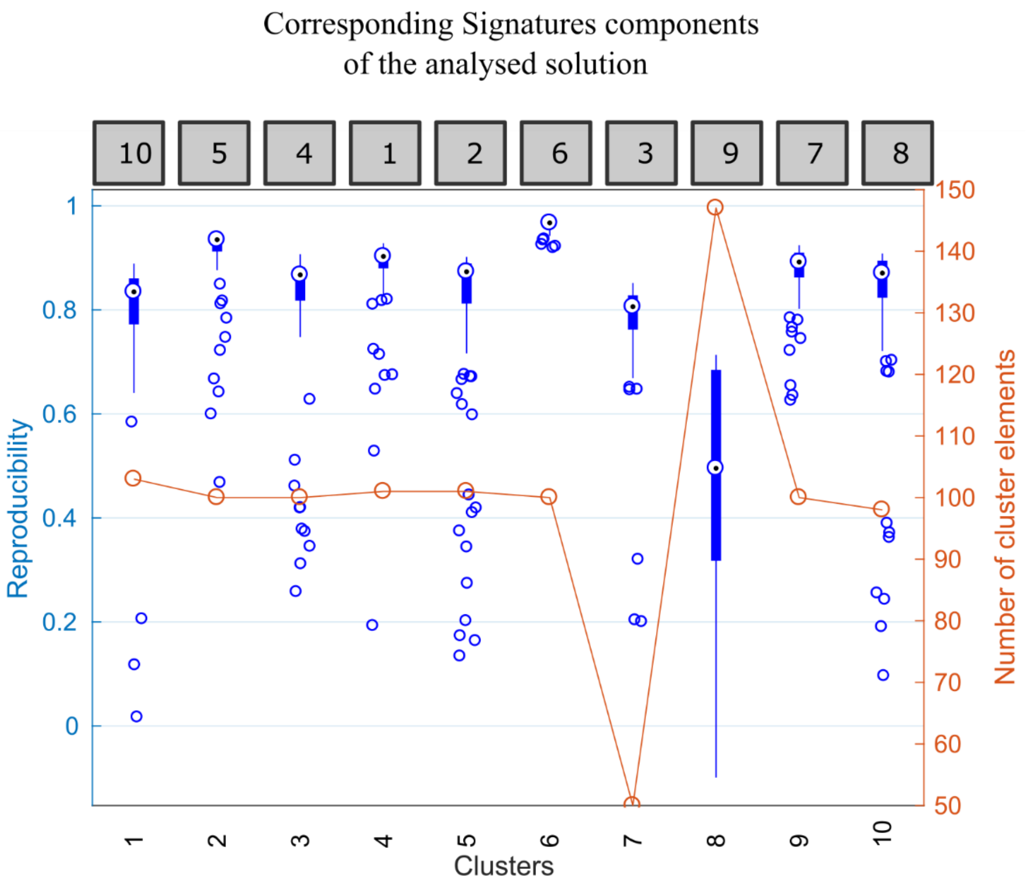

Supplementary Figure 19. Reproducibility analysis of the drug signature components for the best 100 runs out of 10,000 runs of our decomposition algorithm. (left axis) Reproducibility of each k-means clusters measured using the cosine-based silhouette value. The silhouette value for each component is a measure of how similar that component is to the component in its own cluster when compared to the component in other clusters. (right axis) The number of elements in each cluster. Ideally, we would expect 100 components in each cluster.

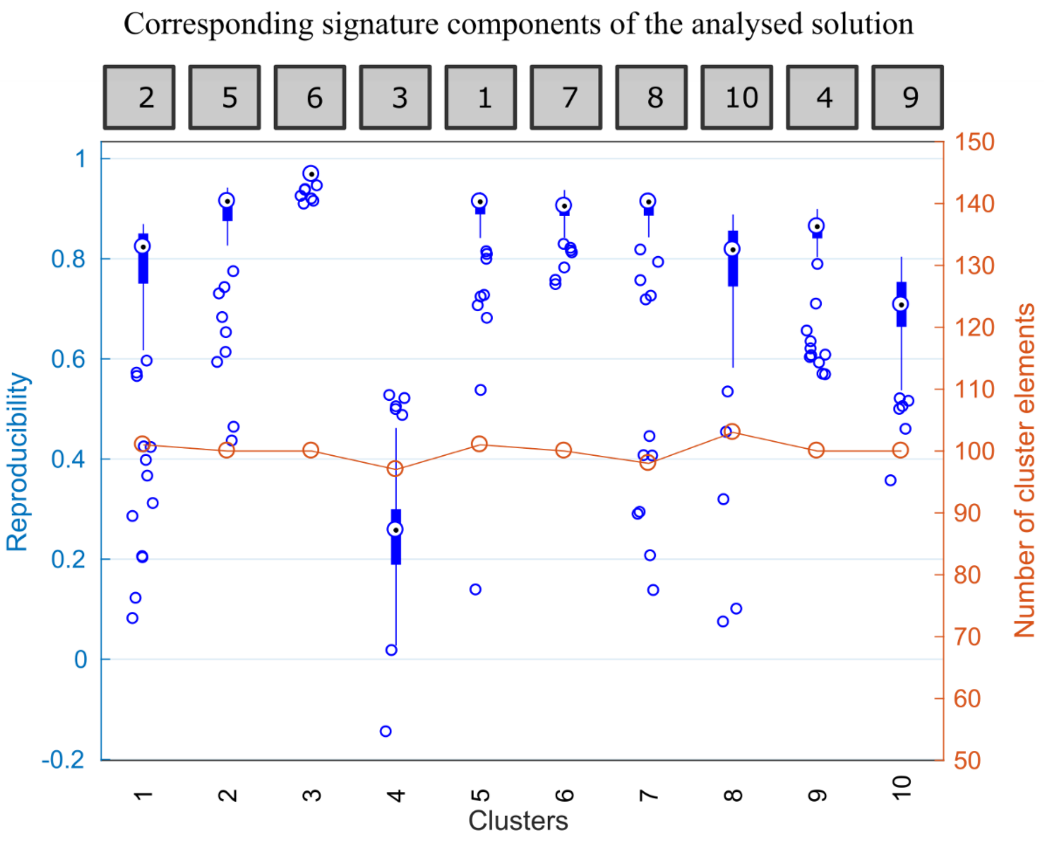

Supplementary Figure 20. Reproducibility analysis of the side effect signatures components for the best 100 runs out of 10,000 runs of our decomposition algorithm. (left axis) Reproducibility of each k-means clusters measured using the cosine-based silhouette value. The silhouette value for each component is a measure of how similar that component is to the component in its own cluster when compared to the component in other clusters. (right axis) The number of elements in each cluster. Ideally, we would expect 100 components in each cluster.

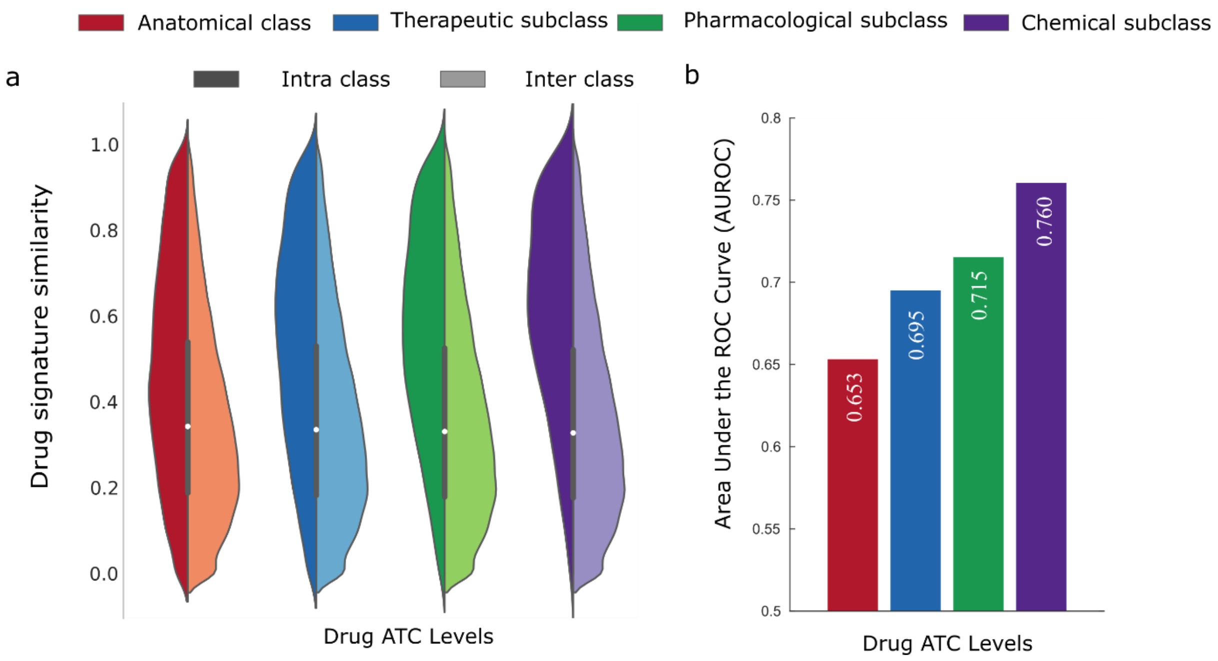

Supplementary Figure 21. Predicting shared drug clinical activity using drug signature similarities. (a) Comparative distribution of drug signature similarity for the intraclass (dark colour), *i.e.* pairs that share ATC category, versus the interclass (light colour), i.e. pairs not known to share ATC in the indicated level. **(b)** Area Under the Receiver Operating Characteristic Curve (AUROC) representing the ability of the drug signature similarity to predict whether which pairs of drugs share Anatomical, Therapeutic and Chemical (ATC) category for each of the different levels of drug activity in the taxonomy: anatomical (red), therapeutic (blue), pharmacological (green) and chemical (purple).

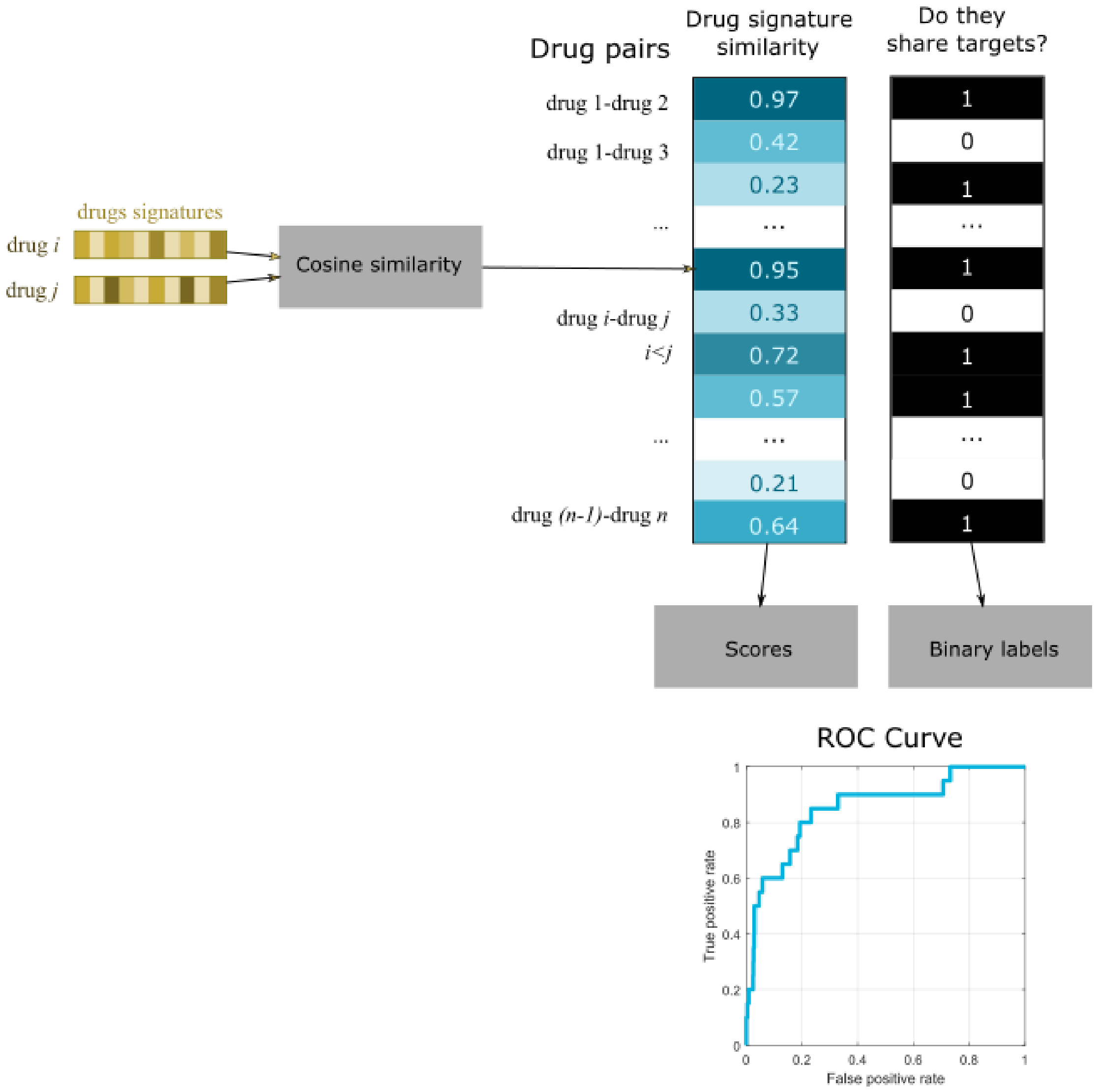

Supplementary Figure 22. Illustrative diagram of our binary classification pipeline to investigate whether drug signature similarities can predict share targets between drugs. After computing the drug signature similarities for all the drug pairs, we build a table containing all these real numbers. Then, we build a table with the same size but containing the binary information to whether the same pairs of drugs are known to share protein targets. Finally, by using the drug signature similarities as scores and the binary labels, we obtain a Receiver Operating (ROC) Curve.

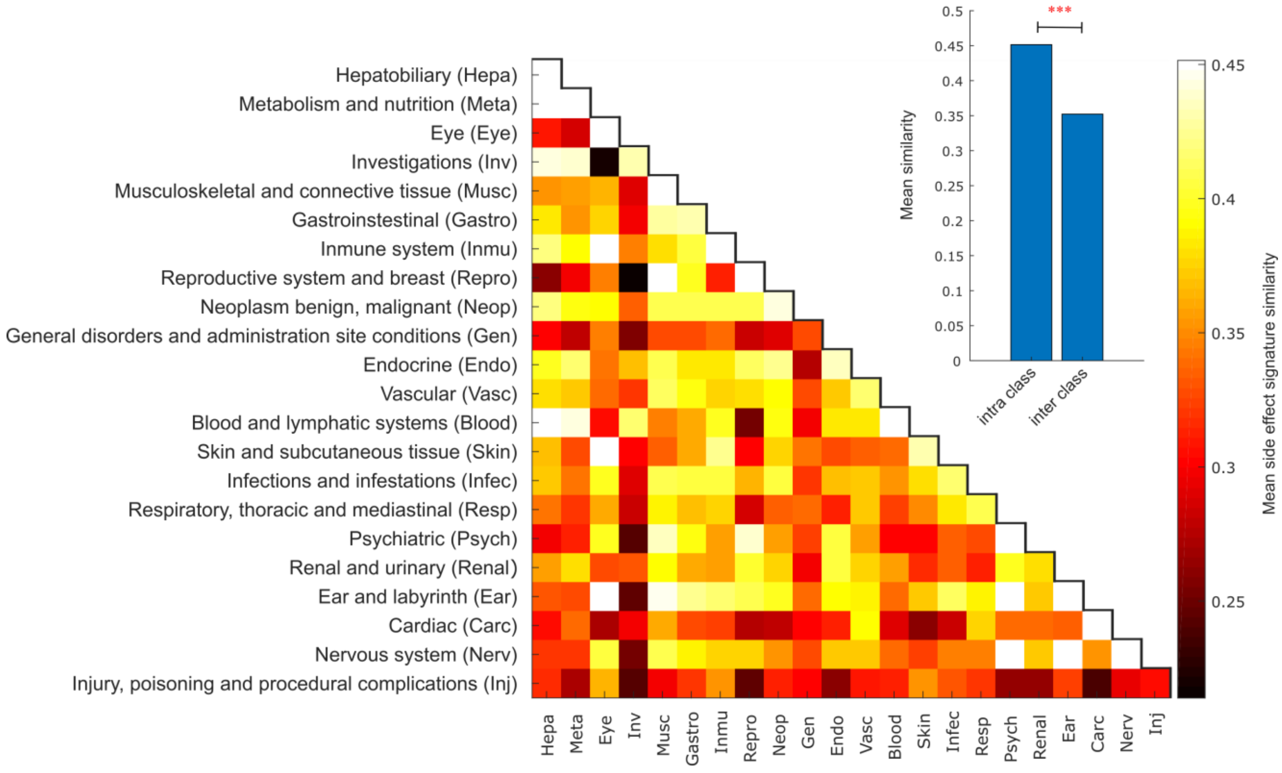

Supplementary Figure 23. Side effect signatures encode side effect phenotypes. Each (x, y) tile represents, for each main Medical Dictionary for Regulatory Activities (MedDRA) classification of disorders, the mean similarity of side effect pairs where one side effect belong to category x and the other to category y. The value ranges from 0.21 (Reproductive systems - Investigations) to 0.58 (Psychiatric – Psychiatric). The colours range between the minimum mean similarity and 0.45, with all values above 0.45 (In the diagonal: 0.49 (Hepa), 0.55 (Eye), 0.57 (Repro), 0.49 (Blood), 0.58 (Psych), 0.54 (Carc), 0.47 (Nerv)) set to 0.45. Inset: the average intra-class similarity is significantly higher than the average inter-class similarity (t-test p-value < 4.37e-16).

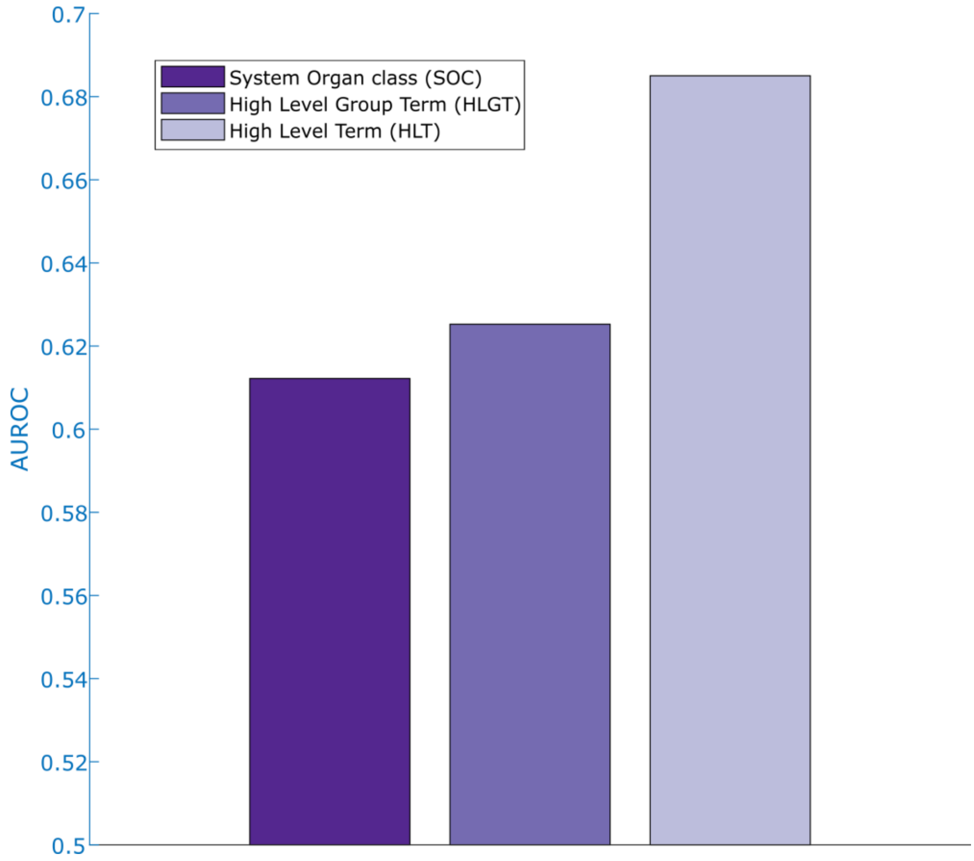

Supplementary Figure 24. Predicting share side effect anatomical/physiological categories for different levels of the MedDRA taxonomy using side effect signatures similarity. Level 1 or System Organ class (SOC): 57,076 side effects that share and 436,445 do not. Level 2 or High-Level Group Term (HLGT): 12,097 shares and 481,424 do not. Level 3 or High-Level Term (HLT): 2,312 shares and 491,209 do not.

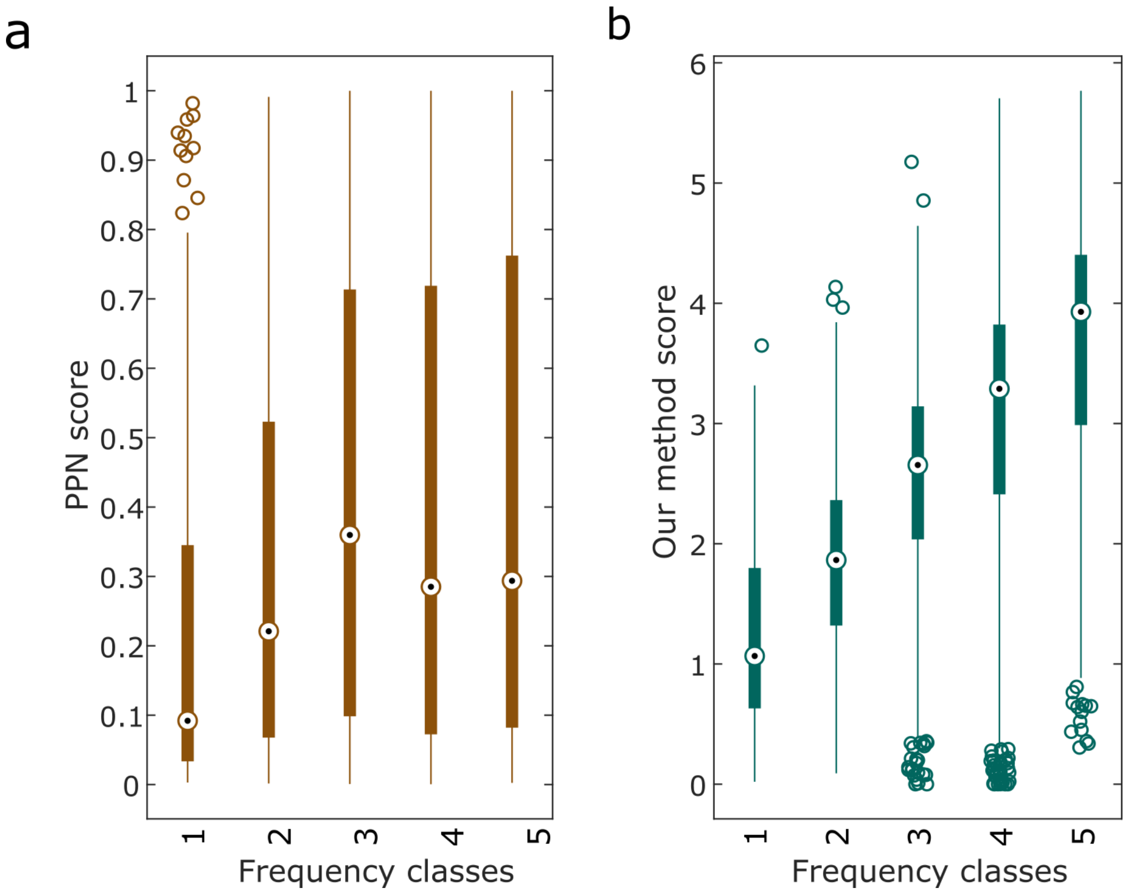

Supplementary Figure 25. Predicted scores by Predictive Pharmacosafety Networks (PPNs) and our method in the held-out test set. (a) Predicted scores by PPNs are weakly correlated to the frequency of the side effect in the population (Pearson correlation ρ=0.08, p < 1.28e-06); (b) Predicted scores by our method are more strongly correlated to the frequency of the side effect in the population (Pearson correlation ρ=0.474, p < 2.394e-209).

Supplementary Tables

Supplementary Table 1. Mapping of frequency values to frequency classes and to frequency values.

Frequency class	Intervention cohort-clinical trials frequency	Assigned value
very frequent	More than 10%	5
frequent	1 to 10%	4
infrequent	0.1 to 1%	3
rare	0.01 to 0.1%	2
very rare	Less than 0.01%	1
zeros*	0%	0
*The zeros are not provided in safety datasets.		

Supplementary Table 2. Summary of therapeutic groups of drugs in our dataset. Drug categories are ordered by numerosity. 676 drugs (89.06%) belongs to one category only, whereas the remaining can belong to more than one category.

Top ATC drug category	Number of drugs	Number of known associations w/ side effect frequency	Average frequency rating value
Nervous system (N)	142	15,470	3.183
Antineoplastic and immunomodulating agents (L)	129	7,286	4.042
Cardiovascular system (C)	111	2,993	3.666
Antiinfectives for systemic use (J)	105	4,135	3.620
Alimentary tract and metabolism (A)	83	2,108	3.660
Genito urinary system and sex hormones (G)	50	1,632	3.706
Sensory organs (S)	47	1,491	3.471
Respiratory system (R)	46	987	3.811
Dermatologicals (D)	45	1,604	3.671
Various (V)	31	609	3.568
Blood and blood-forming organs (B)	31	839	3.639
Musculo squeletal system (M)	30	1,275	3.519
Systemic hormonal preparations, insulins (H)	18	465	3.948
Antiparasitic products, insecticides and repellents (P)	10	235	3.906

Supplementary Table 3. Summary of physiological groups of side effects in our dataset. The categories are ordered by numerosity. Side effect terms could belong to more than one main category. Most side effects belong to one or two categories of disorders (by 59.65%).

Top MedDRA category of disorders	Number of side effects	Number of known associations	Mean frequency
Nervous system disorders	136	5,868	3.527
Skin and subcutaneous tissue disorders	119	4,167	3.378
Gastrointestinal disorders	116	5,913	3.715
Vascular disorders	97	3,725	3.342
Respiratory, thoracic and mediastinal disorders	96	3,604	3.590
General disorders and administration site conditions	92	4,463	3.807
Psychiatric disorders	88	3,409	3.499
Infections and infestations	84	2,964	3.611
Eye disorders	67	1,336	3.206
Reproductive system and breast disorders	61	1,390	3.370
Metabolism and nutrition disorders	60	2,127	3.524
Cardiac disorders	58	2,806	3.469
Injury, poisoning and procedural complications	58	870	3.410
Investigations	57	1,794	3.670
Musculoskeletal and connective tissue disorders	57	2,585	3.570
Renal and urinary disorders	48	1,544	3.240
Blood and lymphatic system disorders	43	1,466	3.283
Immune system disorders	42	1,249	2.611
Neoplasms benign, malignant and unspecified (incl cysts and polyps)	25	206	3.053
Hepatobiliary disorders	24	483	2.687

Endocrine disorders	24	563	3.115
Ear and labyrinth disorders	13	506	3.328
Social circumstances	3	70	3.571
Surgical and medical procedures	2	59	3.271
Pregnancy, puerperium and perinatal conditions	2	13	2.384
Congenital, familial and genetic disorders	1	5	3.400

Supplementary References

1. Kuhn, M., Campillos, M., Letunic, I., Jensen, L. & Bork, P. A side effect resource to capture phenotypic effects of drugs. *Mol Syst Biol* **6**, 343 (2010).

2. Kuhn, M., Letunic, I., Jensen, L. & Bork, P. The SIDER database of drugs and side effects. *Nucleic Acids Res* **44**, D1075–D1079 (2016).

3. Colloca, L. Nocebo effects can make you feel pain. *Science* **358**, 44–44 (2017).

4. Tatonetti, N., Ye, P., Daneshjou, R. & Altman, R. Data-Driven Prediction of Drug Effects and Interactions. *Sci Transl Med* **4**, 125ra31-125ra31 (2012).

5. Banda, J. *et al.* A curated and standardized adverse drug event resource to accelerate drug safety research. *Sci Data* **3**, 160026 (2016).

6. Park, Y. & Marcotte, E. Flaws in evaluation schemes for pair-input computational predictions. *Nat Methods* **9**, 1134–1136 (2012).

7. O'Brien, C. Statistical Learning with Sparsity: The Lasso and Generalizations. *Int Stat Rev* **84**, 156–157 (2016).

8. Koren, Y., Bell, R. & Volinsky, C. Matrix Factorization Techniques for Recommender Systems. *Computer* **42**, 30–37 (2009).

9. Mazumder, R., Hastie, T. & Tibshirani, R. Spectral Regularization Algorithms for Learning Large Incomplete Matrices. *J Mach Learn Res Jmlr* **11**, 2287–2322 (2010).

10. Lee, D. & Seung, H. Learning the parts of objects by non-negative matrix factorization. *Nature* **401**, 788–91 (1999).

11. Lee, D. D. & Seung, H. S. Algorithms for Non-negative Matrix Factorization. in (n.d.).

12. Li, T. & Ding, C. The Relationships Among Various Nonnegative Matrix Factorization Methods for Clustering. *Sixth Int Conf Data Min Icdm'06* 362–371 (2006) doi:10.1109/icdm.2006.160.

13. Shaffer, J. Multiple Hypothesis Testing. *Annu Rev Psychol* **46**, 561–584 (1995).

14. Corsello, S. M. *et al.* The Drug Repurposing Hub: a next-generation drug library and information resource. *Nature Medicine* **23**, 405–408 (2017).
