# Supplementary material for: Predicting the frequencies of drug side effects
Source: Nat Commun. 2020 Sep 11;11:4575. doi: 10.1038/s41467-020-18305-y (PMC7486409; doi:10.1038/s41467-020-18305-y)
Supplement: Supplementary file 2 — Description of Additional Supplementary Files [file 41467_2020_18305_MOESM2_ESM.pdf]

Description of Additional Supplementary Files

File Name: Supplementary Data 1
Description: The standardised drug side effect frequency classes used in our study.

File Name: Supplementary Data 2
Description: The postmarketing drug side effect associations from SIDER and OFFSIDES.

File Name: Supplementary Data 3
Description: Manually curated frequencies of the side effects observed for Semagacestat in clinical trials I, II and III.

File Name: Supplementary Data 4
Description: Monotherapy Anatomical, Therapeutic and Chemical (ATC) code for each drug.

File Name: Supplementary Data 5
Description: Main or High-Level Term (HLT) Medical Dictionary for Regulatory Activities (MedDRA) terminology classification for each side effect term.

File Name: Supplementary Data 6
Description: High-Level Group Term Medical Dictionary for Regulatory Activities (MedDRA) terminology classification for each side effect term.

File Name: Supplementary Data 7
Description: Administration route for each drug.

File Name: Supplementary Data 8
Description: Predicted frequencies of drug side effects together with postmarketing observational evidence from SIDER and OFFSIDES databases. NA stands for no available evidence.

File Name: Supplementary Data 9
Description: Drug signatures that were analysed for biological interpretability.

File Name: Supplementary Data 10
Description: Side effect signatures that were analysed for biological interpretability.

File Name: Supplementary Data 11
Description: Statistically significant associations between the drug signature components and the anatomical level of the ATC hierarchy.

File Name: Supplementary Data 12
Description: Statistically significant associations between the drug signature components and the therapeutic level of the ATC hierarchy.

File Name: Supplementary Data 13
Description: Statistically significant associations between the drug signature components and the pharmacological level of the ATC hierarchy.

File Name: Supplementary Data 14
Description: Statistically significant associations between the side effect signature components and level 1 of the MedDRA hierarchy.

File Name: Supplementary Data 15
Description: Statistically significant associations between the side effect signature components and level 2 of the MedDRA hierarchy.

File Name: Supplementary Data 16
Description: Statistically significant associations between the drug signature components and routes of administration.
