# Supplementary material for: Predicting the frequencies of drug side effects
Source: Nat Commun. 2020 Sep 11;11:4575. doi: 10.1038/s41467-020-18305-y (PMC7486409; doi:10.1038/s41467-020-18305-y)
Supplement: Supplementary file 19 — Reporting Summary [file 41467_2020_18305_MOESM19_ESM.pdf]

nature research

Corresponding author(s): Alberto Paccanaro

Last updated by author(s): Jul 27, 2020

Reporting Summary

Nature Research wishes to improve the reproducibility of the work that we publish. This form provides structure for consistency and transparency in reporting. For further information on Nature Research policies, see our Editorial Policies and the Editorial Policy Checklist.

Statistics

For all statistical analyses, confirm that the following items are present in the figure legend, table legend, main text, or Methods section.

n/a	Confirmed	
☐	☒	The exact sample size (n) for each experimental group/condition, given as a discrete number and unit of measurement
☐	☒	A statement on whether measurements were taken from distinct samples or whether the same sample was measured repeatedly
☐	☒	The statistical test(s) used AND whether they are one- or two-sided<br>*Only common tests should be described solely by name; describe more complex techniques in the Methods section.*
☐	☒	A description of all covariates tested
☐	☒	A description of any assumptions or corrections, such as tests of normality and adjustment for multiple comparisons
☐	☒	A full description of the statistical parameters including central tendency (e.g. means) or other basic estimates (e.g. regression coefficient) AND variation (e.g. standard deviation) or associated estimates of uncertainty (e.g. confidence intervals)
☐	☒	For null hypothesis testing, the test statistic (e.g. F, t, r) with confidence intervals, effect sizes, degrees of freedom and P value noted<br>*Give P values as exact values whenever suitable.*
☐	☒	For Bayesian analysis, information on the choice of priors and Markov chain Monte Carlo settings
☐	☒	For hierarchical and complex designs, identification of the appropriate level for tests and full reporting of outcomes
☐	☒	Estimates of effect sizes (e.g. Cohen's d, Pearson's r), indicating how they were calculated

Our web collection on statistics for biologists contains articles on many of the points above.

Software and code

Policy information about availability of computer code

Data collection	custom code to parse SIDER 4.1 dataset in python 2.7 and jupyter can be found in https://github.com/paccanarolab/Side-effect-Frequencies. This requires data from SIDER 4.1(http://sideeffects.embl.de/) . To obtain drug's ATC codes and routes of administration, we used drug names from DrugBank release 5.0.5 (download date: 17-08-2016) to map to the proprietary data from the World Health Organization (WHO) - ATC index with DDDs 2018 - (https://www.whocc.no/atc_ddd_index/). We also used DrugBank 5.0.5 to obtain drug protein targets and drug Simplified Molecular-Input Line-Entry System (SMILES).
Data analysis	Matlab R2018a code to run our matrix decomposition algorithm can be found at https://github.com/paccanarolab/Side-effect-Frequencies (a copy of this code, together with datasets to reproduce the study can also be found at https://paccanarolab.org/drug-signatures/)

For manuscripts utilizing custom algorithms or software that are central to the research but not yet described in published literature, software must be made available to editors and reviewers. We strongly encourage code deposition in a community repository (e.g. GitHub). See the Nature Research guidelines for submitting code & software for further information.

Data

Policy information about availability of data

All manuscripts must include a data availability statement. This statement should provide the following information, where applicable:

- Accession codes, unique identifiers, or web links for publicly available datasets
- A list of figures that have associated raw data
- A description of any restrictions on data availability

All data necessary to reproduce our work is made available at: https://paccanarolab.org/drug-signatures/

Field-specific reporting

Please select the one below that is the best fit for your research. If you are not sure, read the appropriate sections before making your selection.

☒ Life sciences ☐ Behavioural & social sciences ☐ Ecological, evolutionary & environmental sciences

For a reference copy of the document with all sections, see nature.com/documents/nr-reporting-summary-flat.pdf

Life sciences study design

All studies must disclose on these points even when the disclosure is negative.

Sample size	Our main dataset consists on 759 drugs with 994 side effect terms with ~37K associations. The detailed description of how our sample was obtained can be found in Supplementary Note 1.
Data exclusions	We only considered drugs with known WHO ATC class (v2018), because it was required for the analysis of biological interpretability. We excluded drugs with unknown frequencies of side effects, because our algorithm requires drugs with frequency associations. We also excluded drugs with less than five frequency associations, as these have too little data for training. Complete description of data exclusions can be found in Supplementary Note 1.
Replication	Our entire work is fully reproducible using the code and the dataset provided
Randomization	Randomisation was applied in the selection of the training-test sets in the cross-validation procedure (see Methods)
Blinding	No applicable

Reporting for specific materials, systems and methods

We require information from authors about some types of materials, experimental systems and methods used in many studies. Here, indicate whether each material, system or method listed is relevant to your study. If you are not sure if a list item applies to your research, read the appropriate section before selecting a response.

Materials & experimental systems

n/a	Involved in the study	
☒	☐	Antibodies
☒	☐	Eukaryotic cell lines
☒	☐	Palaeontology and archaeology
☒	☐	Animals and other organisms
☒	☐	Human research participants
☒	☐	Clinical data
☒	☐	Dual use research of concern

Methods

n/a	Involved in the study	
☒	☐	ChIP-seq
☒	☐	Flow cytometry
☒	☐	MRI-based neuroimaging
